# CircC6orf132 Facilitates Proliferation, Migration, Invasion, and Glycolysis of Gastric Cancer Cells Under Hypoxia by Acting on the miR-873-5p/PRKAA1 Axis

**DOI:** 10.3389/fgene.2021.636392

**Published:** 2021-09-30

**Authors:** Weizhi Chen, Yanhong Ji

**Affiliations:** ^1^Department of Pathogenic Biology and Immunology, School of Basic Medical Sciences, Xi’an Jiaotong University Health Science Center, Xi’an, China; ^2^Department of Radiology, The First Affiliated Hospital of Jinzhou Medical University, Jinzhou, China

**Keywords:** CircC6orf132, gastric cancer, glycolysis, miR-873-5p, PRKAA1

## Abstract

**Background:** Hypoxia is a crucial factor in the progression of various tumors, including gastric cancer (GC). Circular RNAs (circRNAs) are important regulators in GC, and this study focused on researching circC6orf132 in GC progression under hypoxia.

**Methods:** *In vitro* experiments were performed in GC cells under hypoxia (1% O_2_). CircC6orf132, microRNA-873-5p (miR-873-5p), and protein kinase AMP-activated alpha 1 catalytic subunit (PRKAA1) levels were examined by real-time polymerase chain reaction (qRT-PCR). Colony formation assay and transwell assay were used for detecting cell proliferation and migration or invasion. Glycolytic metabolism was evaluated using lactate production, glucose uptake, and adenosine triphosphate (ATP) level and extracellular acidification rate (ECAR). Western blotting was performed for determining protein expression. The target interaction was analyzed by dual-luciferase reporter and RNA immunoprecipitation (RIP) assays. *In vivo* assay was conducted *via* mouse xenograft model.

**Results:** The expression of circC6orf132 was significantly high in GC cells under hypoxia. Hypoxia-induced GC proliferation, migration, invasion, and glycolysis were reversed by silencing circC6orf132. CircC6orf132 targeted miR-873-5p; and the inhibition of circC6orf132 knockdown for the effects of hypoxia on GC cells was abrogated by miR-873-5p inhibitor. PRKAA1 was validated as a downstream gene of miR-873-5p, and miR-873-5p functioned as an anticancer molecule in GC cells under hypoxia by downregulating PRKAA1 level. CircC6orf132 could regulate PRKAA1 by sponging miR-873-5p. CircC6orf132/miR-873-5p/PRKAA1 axis could regulate GC progression under the hypoxic condition. CircC6orf132 downregulation reduced tumorigenesis *in vivo* through affecting the miR-873-5p/PRKAA1 axis.

**Conclusion:** CircC6orf132 has been affirmed to promote proliferation, migration, invasion, and glycolysis in GC under hypoxia, partly by depending on the regulation of miR-873-5p/PRKAA1 axis.

## Introduction

Gastric cancer (GC), the fifth most common cancer, is one of the leading causes of cancer-related death around the world (Smyth et al., 2020). Due to the low diagnosis rate of early GC, most GC patients were diagnosed as advanced stage, and the median survival is less than 1 year (Tan, 2019, Smyth et al., 2020). The developmental pathomechanism of GC is complex because GC is a heterogeneous disease resulting from multiple genetic and epigenetic alterations to affect cellular processes and metabolism (Figueiredo et al., 2017). Hypoxia is a significant factor for tumor diffusion and progression and is associated with cancer treatment (Hockel and Vaupel, 2001; Tatum et al., 2006). Hypoxia contributes to tumor glycolytic metabolism that provides energy for tumor proliferation and metastasis (Fang et al., 2008; Tian et al., 2020). To investigate GC pathogenesis under hypoxia is indispensable for early diagnosis and advanced therapy.

Circular RNAs (circRNAs) are produced by “backsplicing” events and presented as specific circular structures, playing important roles in cancer onset and progression (Visci et al., 2020). In addition, circRNAs were also involved in the development of tumors under hypoxia. For instance, circMAT2B enhanced glycolysis and tumor progression of hepatocellular carcinoma under hypoxia by regulating miR-338-3p/PKM2 axis (Li et al., 2019). Ren et al. (2019) found that knockdown of circDENND4C inhibited hypoxia-induced cell metastasis and glycolysis of breast cancer cells *via* upregulating miR-200b and miR-200c. Fang et al. (2020) reported that circSLAMF6 promoted GC cell migration and glycolysis under hypoxic stress through targeting the miR-204-5p/MYH9 axis. The recent circRNA heat-map analysis has indicated that circC6orf132 (hsa_circ_0092341) was highly expressed in GC tissue samples (Liu et al., 2019). However, the function of circC6orf132 in GC progression under hypoxia remains to be explored.

The “sponge mechanism” is the most common functional mechanism of circRNAs in cancers, which can affect microRNA (miRNA) activity to further regulate gene expression (Tang and Hann, 2020). Previous studies have identified the anticancerous response of microRNA-873-5p (miR-873-5p) by targeting the 3′-untranslated regions (3′UTRs) of different genes in many cancers, such as in papillary thyroid cancer and colon cancer (Zhu et al., 2019; Wang et al., 2020). MiR-873-5p was also a tumor inhibitor in GC and long non-coding RNA DDX11-AS1 or TDRG1 accelerated GC development *via* sponging miR-873-5p (Ma et al., 2020; Ren et al., 2020). Protein kinase AMP-activated alpha 1 catalytic subunit (PRKAA1) is a subunit of 5′-AMP-activated protein kinase (AMPK), with the promotion on energy metabolism and tumor progression in GC (Zhang et al., 2019, 2020). Whether circC6orf132 can regulate PRKAA1 by sponging miR-873-5p is unclear in GC.

Herein, the biological influences of circC6orf132 on GC cells under hypoxia were researched. Moreover, we discovered the regulatory network among circC6orf132, miR-873-5p, and PRKAA1. The aim of this study was to explore the role and mechanism of circC6orf132 in regulating hypoxia-induced GC progression.

## Materials and Methods

### Tissue Collection

Surgical resection was performed for 43 GC patients at The First Affiliated Hospital of Jinzhou Medical University. GC tissues (*n* = 43) and normal non-cancerous tissues (*n* = 43) were collected after the surgery and then placed in liquid nitrogen for stable storage. Any therapy has not been used for these GC patients. This research was proceeded based on obtaining the written informed consent forms from all patients and ratification from the Ethics Committee of The First Affiliated Hospital of Jinzhou Medical University.

### Cell Culture and Hypoxia Stimulation

Human gastric epithelial cell line GES-1 cell line and CG cell lines (N87, MKN-45, AGS, and HGC-27) were all provided by Cobioer Biosciences Co., Ltd. (Nanjing, China). Roswell Park Memorial Institute 1,640 (RPMI 1640; Gibco, Carlsbad, CA, United States) was added with 10% fetal bovine serum (FBS; Gibco) and 1% penicillin/streptomycin (Gibco) for preparing cell culture medium. Cells were cultured in the humidified air with 5% CO_2_ and temperature at 37°C. Hypoxia environment was mimicked using a hypoxia chamber with 1% O_2_. AGS and HGC-27 cells were exposed to hypoxia environment for different times (0, 3, 6, 12, 24, and 48 h).

### RNA Isolation, RNase R Treatment, and Quantitative Real-Time Polymerase Chain Reaction

Total RNA of collected tissues and cells was purified by RNAiso Plus Reagent (Takara, Shiga, Japan). In addition, cytoplasmic and nuclear RNA was, respectively isolated from AGS and HGC-27 cells by PARIS^TM^ Kit (Invitrogen, Carlsbad, CA, United States). Ribonuclease R (RNase R; Epicenter Technologies, Madison, WI, United States) was treated to total RNA with 3 U/μg for 1 h to assess molecular stability of circC6orf132. The complementary DNA (cDNA) was synthesized using PrimeScript^TM^ RT Master Mix (Takara), and the expression of each molecule was detected by TB Green^®^ *Premix Ex Taq*^TM^ II reagent (Takara) through the specific primers. The sequences of forward (F) and reverse (R) primers were as follows: circC6orf132 (F, 5′-TCCTGACCTCCCTCCTGAC-3′ and R, 5′-TGTTTTCTCTTGACGGATGG-3′); C6orf132 (F, 5′-AGTGCC CATTTCAGAGGAGC-3′ and R, 5′-TGGCTGGCCTTGGTA ATCTG-3′); miR-873-5p (F, 5′-GGGGCAGGAACTTGTGAG-3′ and R, 5′-GTGTGGTGTGGTATGGTGTG-3′); PRKAA1 (F, 5′-TATGGCAGAAGTATGTAGAG-3′ and R, 5′-ACGGAAATCCA GTAGATAAG-3′); β-actin (F: 5′-TTCGAGCAAGAGATGGC CA-3′ and R: 5′-TACATGGTGGTGCCGCC-3′); U6 (F: 5′-CTC GCTTCGGCAGCACA-3′ and R: 5′-AACGCTTCACGAATT TGCGT-3′). The normalization of expression level was performed with β-actin and U6 as the housekeeping genes. The comparative cycle threshold (2^–ΔΔCt^) method was used to analyze the relative expression level.

### Transient and Stable Transfection

Small interfering RNAs for circC6orf132 and negative control (si-circC6orf132 and si-NC), miR-873-5p mimic and negative control (miR-873-5p and miR-NC), and miR-873-5p inhibitor and negative control (in-miR-873-5p and in-miR-NC) were obtained from GenePharma (Shanghai, China). The pCd5-ciR-circC6orf132 (circC6orf132) and pcDNA-PRKAA1 (PRKAA1) vectors were constructed by inserting the sequence of circC6orf132 and PRKAA1 into pCD5-ciR (Geneseed, Guangzhou, China) and pcDNA (Invitrogen), respectively. Then these oligonucleotides and vectors were transiently transfected into AGS and HGC-27 cells using Lipofectamine^TM^3000 Reagent (Invitrogen). For stable transfection, lentiviral vectors containing short hairpin RNA for circC6orf132 and negative control (sh-circC6orf132 and sh-NC) were bought from GenePharma.

### Colony Formation Assay

After transfection for 24 h, cells were cultured under hypoxia for 48 h. The untransfected cells were also cultured under hypoxia or normoxia for 48 h. Five hundred collected AGS or HGC-27 cells were transplanted into the six-well plates and cultivated for 2 weeks. The colony spots were stained by crystal violet (Sigma-Aldrich, St. Louis, MO, United States) after fixation in 4% paraformaldehyde (Sigma-Aldrich), followed by counting the number of purple colonies.

### Transwell Assay

The migratory and invasive abilities of AGS and HGC-27 cells were assessed by transwell assay with 24-well transwell chambers (Corning Inc., Corning, NY, United States). Especially, the chamber for invasion detection was required to be covered with Matrigel in the upper chamber (Corning Inc.). The upper and lower chambers were, respectively added with cell suspension in serum-free medium and RPMI 1640 medium containing 10% FBS. After 24 h, cells migrated and invaded into the lower chamber were fastened and stained using 4% paraformaldehyde and crystal violet (Sigma-Aldrich). Through × 100 magnification by the inverted microscope (Olympus, Tokyo, Japan), cell photographing and counting were performed.

### Glycolytic Analysis

Cell medium was harvested after transfection and treatment of AGS and HGC-27 cells. The concentrations of lactate and glucose were, respectively examined using a Lactate Assay Kit and Glucose Assay Kit (Sigma-Aldrich). In addition, adenosine triphosphate (ATP) level was measured by an ATP Assay Kit (Sigma-Aldrich). The concentration of glucose or lactate and ATP level were normalized to total protein concentration.

### Extracellular Acidification Rate

Extracellular acidification rate (ECAR) was measured using Seahorse Extracellular Flux Analyzer XF96 (Seahorse Bioscience, Billerica, MA, United States) according to the manufacturer’s guidelines. Briefly, 2 × 10^4^ cells/well were seeded into the XF96-well plates for 18 h. Then cell medium was replaced with XF assay unbuffered medium supplemented with 2 mM of glutamine, followed by the incubation of 10 mM of glucose (Glc), 1 μM of oligomycin (Oligo), and 80 mM of 2-deoxyglucose (2-DG). ECAR (mpH/min) was acquired under the analyzer.

### Western Blotting

Protein denaturation was performed at 100°C after extraction from tissues or cells by radioimmunoprecipitation assay (RIPA; Thermo Fisher Scientific, Waltham, MA, United States); then protein products were used for sodium dodecyl sulfate–polyacrylamide gel electrophoresis (SDS-PAGE). After protein transferring and non-specific binding prevention, the polyvinylidene fluoride membranes (Thermo Fisher Scientific) were incubated with primary antibodies for hypoxia-inducible factor 1α (HIF1α; ab179483, 1:1,000), glucose transporter 1 (GLUT1; ab128033, 1:1,000), hexokinase II (HK2; ab227198, 1:1,000), PRKAA1 (ab32047, 1:1,000), and β-actin (ab5694, 1:3,000) at 4°C overnight. Subsequently, the incubation of Goat Anti-Rabbit IgG H&L [horseradish peroxidase (HRP)] second antibody (ab205718, 1:5,000) was performed for 1 h at the room temperature. Novex^TM^ ECL Chemiluminescent Substrate Reagent Kit (Invitrogen) was applied for the presence of protein bands, and level analysis was performed in Image Lab software (Bio-Rad, Hercules, CA, United States).

### Dual-Luciferase Reporter Assay

Wild-type (WT) luciferase vectors for circC6orf132 or PRKAA1 3′UTR (circC6orf132 WT and PRKAA1 3′UTR WT) containing the binding sites of miR-873-5p and their mutant controls (circC6orf132 MUT and PRKAA1 3′UTR MUT) were constructed using the basic luciferase vector psiCHECK-2 (Promega, Madison, WI, United States). AGS and HGC-27 cells were cultured overnight and transfected with every constructed vector and miR-873-5p or miR-NC. At 48 h post-transfection, the luciferase intensity was determined through the dual-luciferase reporter system (Promega).

### RNA Immunoprecipitation Assay

The potential interaction between circC6orf132 and miR-873-5p was validated by RIPA using Magna RIP RNA-Binding Protein Immunoprecipitation Kit (Millipore, Billerica, MA, United States). AGS and HGC-27 cells were collected and lysed in RIP buffer, followed by overnight incubation in magnetic beads coated with antibodies of Argonaute 2 (Ago2) or immunoglobulin G (IgG) at 4°C. RNA binding to magnetic beads was extracted, and the circC6orf132 or miR-873-5p expression was assayed *via* qRT-PCR.

### Tumor Xenograft Assay

Five-week-old male BALB/c nude mice (Vital River Laboratory Animal Technology, Beijing, China) were subcutaneously injected with 1 × 10^6^ sh-circC6orf132 or sh-NC transfected HGC-27 cells (*n* = 5 per group). Tumor size was monitored by digital caliper once a week, and tumor volume was calculated according to the following formula: tumor length × width^2^/2. All mice were euthanatized after 28 days, and tumors were excised. After the measurement of tumor weight, the tissues were used for isolation of total RNA or protein. The qRT-PCR and Western blotting were performed to detect the RNA or protein level. Lactate production and glucose uptake in serum samples of mice were detected using the corresponding kits. The glycolytic markers (GLUT1; HK2) and proliferation-related proteins [Ki-67; proliferating cell nuclear antigen (PCNA)] were determined using immunohistochemical (IHC) analysis. This assay was authorized by the Animal Ethical Committee of The First Affiliated Hospital of Jinzhou Medical University, and the Guidelines for Laboratory Animals of the National Institutes of Health (NIH) were followed.

### Statistical Analysis

Data were expressed as the mean ± standard deviation (SD) and processed by GraphPad Prism 7.0. Linear association was analyzed using Pearson’s correlation coefficient. Statistical analysis was performed in SPSS 24.0, and the difference was compared by Student’s *t*-test in two groups, as well as one-way analysis of variance (ANOVA) followed by Tukey’s test in multiple groups. The difference was statistically significant with *p* < 0.05.

## Results

### CircC6orf132 Was Upregulated in Gastric Cancer Cells Under Hypoxia

**FIGURE 1:**
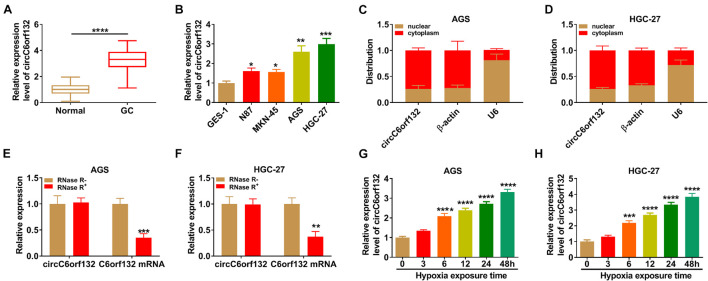
CircC6orf132 was upregulated in gastric cancer (GC) cells under hypoxia. **(A,B)** The qRT-PCR was performed for the determination of circC6orf132 in GC/normal tissues **(A)** and cells **(B)**. **(C,D)** The circC6orf132, β-actin, and U6 levels were measured by qRT-PCR after nuclear and cytoplasmic RNA isolation from AGS and HGC-27 cells. **(E,F)** The detection of circC6orf132 and C6orf132 was conducted using qRT-PCR after RNase R treatment. **(G,H)** The circC6orf132 expression was assayed *via* qRT-PCR after hypoxia exposure for 0, 3, 6, 12, 24, or 48 h in AGS and HGC-27 cells. *p < 0.05, **p < 0.01, ***p < 0.001, and ****p < 0.0001.

The expression of circC6orf132 in GC was assayed using qRT-PCR. As exhibited in Figures 1A,B, there was the upregulation of circC6orf132 in GC tissue samples and cell lines (N87, MKN-45, AGS, and HGC-27) by comparison with normal tissue samples and GES-1 cell line. The following experiments were performed in AGS and HGC-27 cells with higher circC6orf132 level (than N87 and MKN-45 cells). Then cell localization was analyzed by qRT-PCR after isolation of nuclear and cytoplasmic RNA, with β-actin (for cytoplasm) and U6 (for nucleus) as the control genes. As shown in Figures 1C,D, circC6orf132 was mainly localized in the cytoplasm of AGS and HGC-27 cells. After RNase R digestion and qRT-PCR detection, we found that circC6orf132 was more stable than linear C6orf132 in GC cells (Figures 1E,F). Furthermore, the qRT-PCR was performed to assess whether circC6orf132 was dysregulated under the hypoxic condition. The Western blotting data have shown that HIF1α protein expression was significantly upregulated in hypoxia group relative to normoxia group, suggesting that the hypoxia environment was efficient (Supplementary Figure 1). The qRT-PCR results indicated that circC6orf132 level was increased in a time-dependent manner after AGS and HGC-27 cells were exposed to hypoxia (Figures 1G,H). CircC6orf132 upregulation was also found in GC cells under hypoxia.

### Silencing circC6orf132 Impeded Proliferation, Migration, Invasion, and Glycolysis of Gastric Cancer Cells Under Hypoxia

**FIGURE 2:**
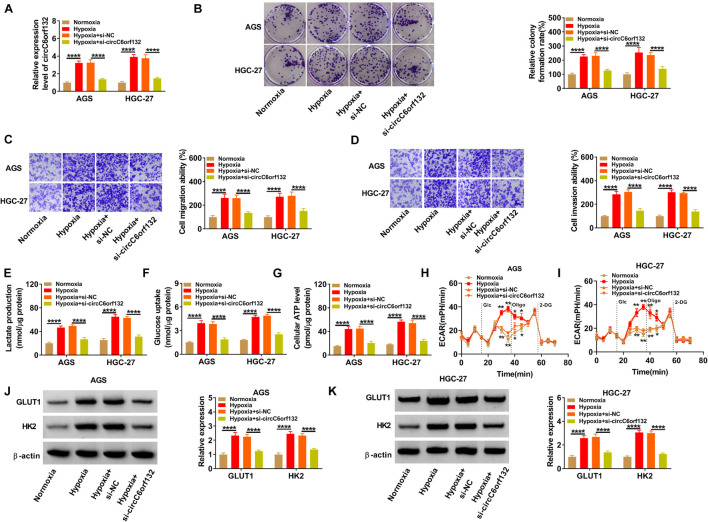
Silencing circC6orf132 impeded proliferation, migration, invasion, and glycolysis of gastric cancer (GC) cells under hypoxia. AGS and HGC-27 cells were transfected with si-NC or si-circC6orf132 under hypoxia for 48 h, and untransfected cells were cultured under normoxia or hypoxia for 48 h. **(A)** The level of circC6orf132 was examined using qRT-PCR. **(B–D)** Colony formation assay and transwell assay were, respectively, used to assess cell proliferation **(B)** and migration or invasion **(C,D)**. **(E–I)** Glycolysis was evaluated by detecting lactate production **(E)**, glucose uptake **(F)**, adenosine triphosphate (ATP) level **(G)**, and extracellular acidification rate (ECAR) **(H,I)**. **(J,K)** GLUT1 and HK2 protein levels were measured by Western blotting. *p < 0.05, **p < 0.01, and ****p < 0.0001.

AGS and HGC-27 cells were transfected with si-NC or si-circC6orf132, followed by hypoxia treatment for 48 h. The result of qRT-PCR demonstrated that transfection of si-circC6orf132 inhibited this upregulation of circC6orf132 by hypoxia (Figure 2A). By performing colony formation assay and transwell assay, we found that hypoxia resulted in the stimulative effects on cell proliferation (Figure 2B), migration (Figure 2C), and invasion (Figure 2D), while subsequent circC6orf132 knockdown rescued these effects. Glycolytic metabolism was assessed by detecting some indexes and associated genes in the process of glycolysis. The lactate production (Figure 2E), glucose uptake (Figure 2F), and ATP level (Figure 2G) were all promoted in hypoxia group compared with normoxia group, which was balanced out following the downregulation of circC6orf132. Transfection of si-circC6orf132 also attenuated the hypoxia-induced increase of ECAR in AGS and HGC-27 cells, confirming that the silence of circC6orf132 repressed glycolysis in hypoxic GC cells (Figures 2H,I). Western blotting also suggested that the protein level upregulation of GLUT1 and HK2 in hypoxia group was counteracted in hypoxia + si-circC6orf132 group of AGS and HGC-27 cells (Figures 2J,K). In addition, circC6orf132 upregulation has aggravated the hypoxia-induced proliferation, migration, invasion, and glycolysis in AGS and HGC-27 cells (Supplementary Figure 2). Taken together, circC6orf132 significantly promoted GC progression under hypoxia.

### CircC6orf132 Interacted With miR-873-5p

**FIGURE 3:**
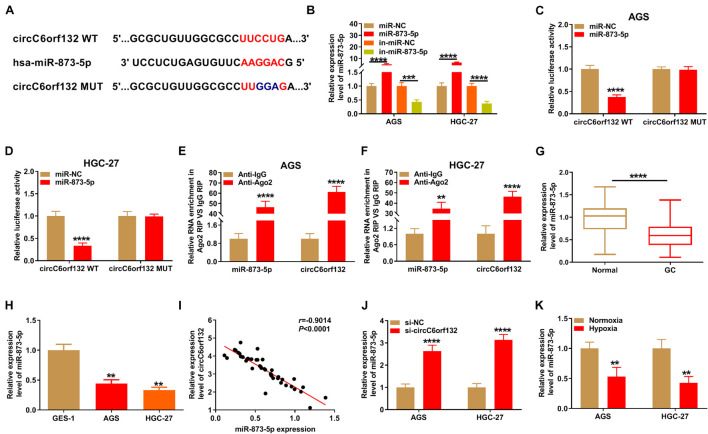
CircC6orf132 interacted with miR-873-5p. **(A)** The CircInteractome was applied for predicting the binding sites between circC6orf132 and miR-873-5p. **(B)** Transfection efficiencies of miR-873-5p mimic and in-miR-873-5p were estimated by qRT-PCR. (C–F) Dual-luciferase reporter assay **(C,D)** and RNA immunoprecipitation (RIP) assay **(E,F)** were used to analyze whether circC6orf132 could interact with miR-873-5p. **(G,H)** The analysis of miR-873-5p expression in gastric cancer (GC) tissues **(G)** and cells **(H)** was carried out applying with qRT-PCR. **(I)** The linear relationship between circC6orf132 and miR-873-5p was analyzed through Pearson’s correlation coefficient. **(J,K)** The miR-873-5p detection was performed *via* qRT-PCR in si-NC or si-circC6orf132 transfection group **(J)** and hypoxia or normoxia treatment group **(K)** of AGS and HGC-27 cells. **p < 0.01, ***p < 0.001, and ****p < 0.0001.

CircInteractome software was used to search for circC6orf132-associated miRNAs. As Figure 3A illustrates, the complementary miR-873-5p binding sites were presented in circC6orf132 sequence. Then dual-luciferase reporter assay was carried out by co-transfection of circC6orf132 WT or circC6orf132 MUT and miR-873-5p or miR-NC. The overexpressed efficiency of miR-873-5p mimic and silencing effectivity of in-miR-873-5p were excellent in AGS and HGC-27 cells relative to miR-NC or in-miR-NC transfection (Figure 3B). Relative luciferase activity of circC6orf132 WT group was significantly decreased by overexpression of miR-873-5p, but that of circC6orf132 MUT group was impervious in AGS and HGC-27 cells (Figures 3C,D). The large enrichment of miR-873-5p and circC6orf132 in Ago2 group (contrasted to IgG group) also indicated the potential combination between circC6orf132 and miR-873-5p (Figures 3E,F). In comparison with normal tissues and GES-1 cells, the level of miR-873-5p was downregulated in GC tissues (Figure 3G) and AGS/HGC-27 cells (Figure 3H). Meanwhile, the absolute quantification indicated that copy number of circC6orf132 was much higher than that of miR-873-5p in GC samples (Supplementary Figure 3). It was conspicuous that circC6orf132 expression was negatively related to miR-873-5p (*r* = −0.9014, *p* < 0.0001) in GC tissue samples (Figure 3I), and knockdown of circC6orf132 motivated the expression of miR-873-5p in GC cells (Figure 3J). In addition, the miR-873-5p expression was lower in hypoxia group of AGS and HGC-27 cells than that in normoxia group (Figure 3K). All these results identified miR-873-5p as a target of circC6orf132.

### CircC6orf132 Affected Biological Processes of Gastric Cancer Cells Under Hypoxia *via* Sponging miR-873-5p

**FIGURE 4:**
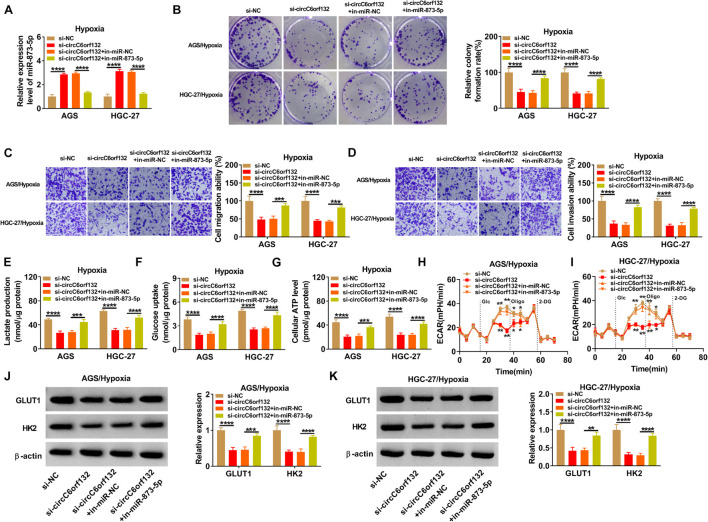
CircC6orf132 affected biological processes of gastric cancer (GC) cells under hypoxia *via* sponging miR-873-5p. AGS and HGC-27 cells were transfected with si-NC, si-circC6orf132, si-circC6orf132 + in-miR-NC, or si-circC6orf132 + in-miR-873-5p under hypoxia for 48 h. **(A)** The qRT-PCR was employed for assaying miR-873-5p expression. **(B–D)** Cell proliferation **(B)** and migration or invasion **(C,D)** examination was performed by colony formation assay and transwell assay. **(E–I)** Lactate production **(E)**, glucose uptake **(F)**, adenosine triphosphate (ATP) level **(G)**, and extracellular acidification rate (ECAR) **(H,I)** were detected to analyze the glycolytic metabolism. **(J,K)** The measurement of GLUT1 and HK2 protein expression was completed using Western blotting. *p < 0.05, **p < 0.01, ***p < 0.001, and ****p < 0.0001.

Transfection of si-circC6orf132, si-circC6orf132 + in-miR-873-5p or the matched negative control was performed in AGS and HGC-27 cells under hypoxia for 48 h. The introduction of in-miR-873-5p reduced the high expression of miR-873-5p by si-circC6orf132, showing that the inhibition of miR-873-5p was successful through in-miR-873-5p transfection (Figure 4A). As the consequences of miR-873-5p downregulation, si-circC6orf132-mediated repression of proliferation (Figure 4B), migration (Figure 4C), and invasion (Figure 4D) in AGS and HGC-27 cells under hypoxia were returned. The same reversal of in-miR-873-5p for si-circC6orf132 was also found in glycolytic metabolism. Lactate production (Figure 4E), glucose uptake (Figure 4F), ATP level (Figure 4G), ECAR (Figures 4H,I), and GLUT1/HK2 protein levels (Figures 4J,K) were much higher in si-circC6orf132 + in-miR-873-5p group than in alone si-circC6orf132 group. Thus, the regulation of circC6orf132 in GC cell progression under hypoxia was partly achieved by targeting miR-873-5p.

### PRKAA1 Was Regulated by circC6orf132/miR-873-5p Axis

**FIGURE 5:**
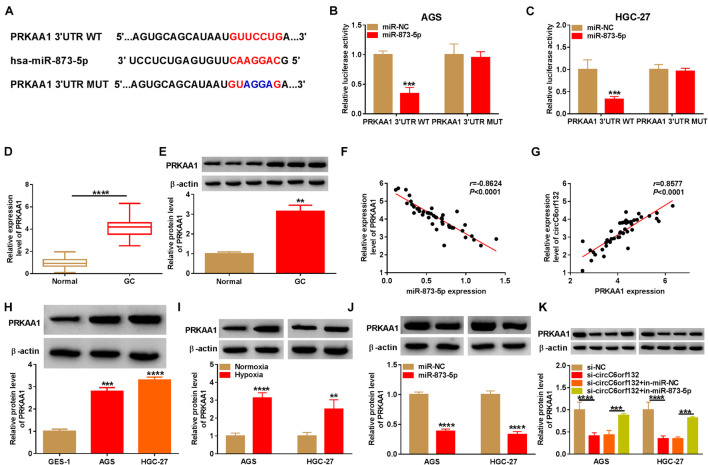
PRKAA1 was regulated by circC6orf132/miR-873-5p axis. **(A)** The binding sites of miR-873-5p and PRKAA1 3′UTR were shown by TargetScan. **(B,C)** The combination between miR-873-5p and PRKAA1 3′UTR was verified in AGS and HGC-27 cells using the dual-luciferase reporter assay. **(D,E)** The mRNA and protein levels of PRKAA1 in gastric cancer (GC) and normal tissues were determined by qRT-PCR and Western blotting. **(F,G)** Pearson’s correlation coefficient was used to analyze the relation between PRKAA1 and miR-873-5p **(F)** or circC6orf132 **(G)**. **(H,I)** Western blotting was exploited for protein analysis of PRKAA1 in GC cells **(H)** and GC cells under normoxia or hypoxia **(I)**. **(J,K)** The protein expression of PRKAA1 was detected with Western blotting in AGS and HGC-27 cells transfected with miR-NC or miR-873-5p **(J)** and si-circC6orf132, si-circC6orf132 + in-miR-873-5p or the negative controls **(K)**. **p < 0.01, ***p < 0.001, and ****p < 0.0001.

The target genes of miR-873-5p were sought by TargetScan, and PRKAA1 was predicted to bind to miR-873-5p in nucleotide sites (Figure 5A). The binding between miR-873-5p and PRKAA1 was also affirmed by dual-luciferase reporter assay due to the luciferase signal inhibition of miR-873-5p overexpression on PRKAA1 3′UTR WT group rather than PRKAA1 3′UTR MUT group (Figures 5B,C). The qRT-PCR and Western blotting exhibited that PRKAA1 mRNA and protein levels were overexpressed in GC samples by contrast with normal control samples (Figures 5D,E). Through the linear analysis in GC tissues, we found a negative relation (*r* = −0.8624, *p* < 0.0001) between miR-873-5p and PRKAA1 (Figure 5F) but a positive relation (*r* = 0.8577, *p* < 0.0001) between circC6orf132 and PRKAA1 (Figure 5G). Upregulation of PRKAA1 protein expression was also indicated in AGS and HGC-27 cells (Figure 5H) and hypoxia-treated cells (Figure 5I). The effects of miR-873-5p and circC6orf132 on PRKAA1 expression were analyzed by Western blotting. The results revealed the direct downregulation of PRKAA1 by miR-873-5p overexpression (Figure 5J) and the indirect inhibition of PRKAA1 by si-circC6orf132 *via* increasing miR-873-5p (Figure 5K). CircC6orf132 could target miR-873-5p to regulate PRKAA1 positively.

### MiR-873-5p Suppressed the Progression of Gastric Cancer Under Hypoxia by Downregulating PRKAA1

**FIGURE 6:**
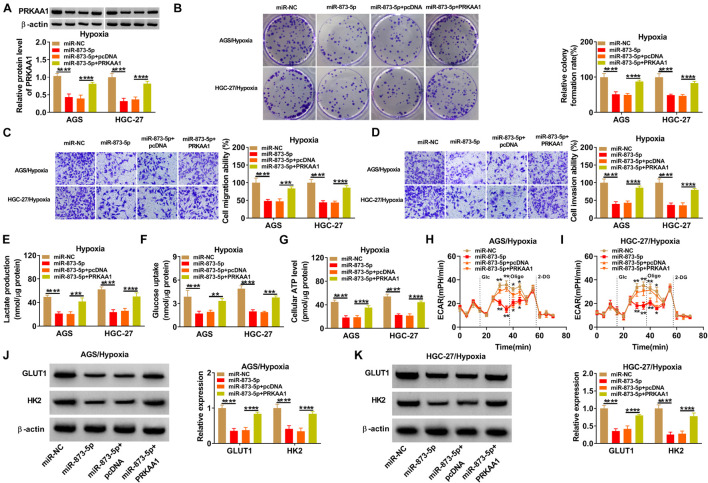
MiR-873-5p suppressed the progression of gastric cancer (GC) under hypoxia by downregulating PRKAA1. AGS and HGC-27 cells were transfected with miR-NC, miR-873-5p, miR-873-5p + pcDNA, or miR-873-5p + PRKAA1 under hypoxia for 48 h. **(A)** PRKAA1 protein expression was analyzed by Western blotting. **(B–D)** The evaluation of cellular ability was implemented using colony formation assay for proliferation **(B)** and transwell assay for migration or invasion **(C,D)**. **(E–K)** The assessment of glycolytic process was according to lactate production **(E)**, glucose uptake **(F)**, adenosine triphosphate (ATP) level **(G)**, extracellular acidification rate (ECAR) **(H,I)**, and GLUT1 or HK2 protein levels **(J,K)**. *p < 0.05, **p < 0.01, ***p < 0.001, and ****p < 0.0001.

The mechanism between miR-873-5p and PRKAA1 in GC was also explored by reverted transfection under hypoxia in AGS and HGC-27 cells. PRKAA1 vector greatly promoted PRKAA1 level because PRKAA1 transfection mitigated the miR-873-5p-induced PRKAA1 protein inhibition (Figure 6A). The high miR-873-5p expression incurred proliferation suppression (Figure 6B) and migration or invasion retardment (Figures 6C,D) under hypoxia, whereas these influences were ameliorated by increasing PRKAA1 expression in AGS and HGC-27 cells. The upregulation of PRKAA1 also abolished the blockage of glycolytic process caused by miR-873-5p after the detection of lactate production (Figure 6E), glucose uptake (Figure 6F), ATP level (Figure 6G), ECAR (Figures 6H,I), and GLUT1/HK2 protein levels (Figures 6J,K). The above evidence supported miR-873-5p as an inhibitor in GC development under hypoxia by targeting PRKAA1.

### Downregulation of circC6orf132 Inhibited Tumor Progression *in vitro* and *in vivo* by the miR-873-5p/PRKAA1 Axis

**FIGURE 7:**
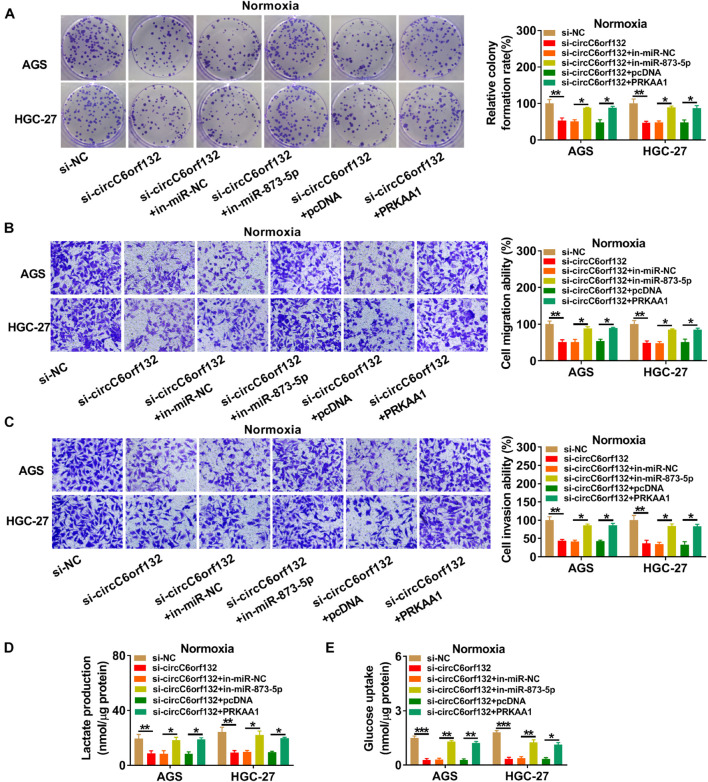
Knockdown of circC6orf132 suppressed the progression of normoxic gastric cancer (GC) cells by regulating the miR-873-5p/PRKAA1 axis. AGS and HGC-27 cells were transfected with si-NC, si-circC6orf132, si-circC6orf132 + in-miR-NC, si-circC6orf132 + in-miR-873-5p, si-circC6orf132 + pcDNA, and si-circC6orf132 + PRKAA1 for 48 h. **(A)** Colony formation assay was performed for proliferation analysis. **(B,C)** Transwell assay was conducted for the determination of migration **(B)** and invasion **(C)**. **(D,E)** Lactate production **(D)** and glucose uptake **(E)** were examined for the evaluation of glycolysis. ^∗^*p* < 0.05, ^∗∗^*p* < 0.01, and ^∗∗∗^*p* < 0.001.

**FIGURE 8:**
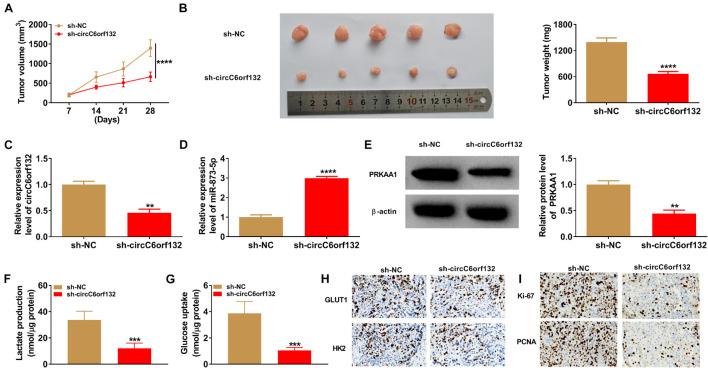
Downregulation of circC6orf132 inhibited tumor growth *in vivo* by the miR-873-5p/PRKAA1 axis. **(A)** Tumor volume was measured weekly after cell injection of sh-NC or sh-circC6orf132 group. **(B)** Tumors were weighed after excision from mice. **(C–E)** The qRT-PCR and Western blotting were used to examine the circC6orf132 or miR-873-5p expression **(C,D)** and PRKAA1 protein level **(E)**. **(F,G)** Lactate production **(F)** and glucose uptake **(G)** in tumor tissues were determined by the commercial kits. **(H,I)** The protein levels of glycolytic markers **(H)** and proliferation-related proteins **(I)** were analyzed using immunohistochemical (IHC) assay. ^∗∗^*p* < 0.01, ^∗∗∗^*p* < 0.001, and ^****^p < 0.0001.

The experimental results have revealed that knockdown of circC6orf132 also suppressed the malignant behaviors of normoxic AGS and HGC-27 cells, and miR-873-5p inhibition or PRKAA1 overexpression relived the effects of si-circC6orf132 on normoxic cells (Figure 7). Thus, circC6orf132/miR-873-5p/PRKAA1 axis was also affirmed in the progression of GC under normoxia. Furthermore, circC6orf132 was researched by xenograft models in mice. After cell injection for 28 days, tumor volume and weight were markedly repressed in sh-circC6orf132 group with comparison with sh-NC group (Figures 8A,B). The qRT-PCR indicated that the level of circC6orf132 in sh-circC6orf132 group was lower than that in sh-NC group (Figure 8C). The knockdown of circC6orf132 facilitated the miR-873-5p expression (Figure 8D) but downregulated the protein level of PRKAA1 (Figure 8E) after qRT-PCR and Western blotting detection in tumor tissues. To explore whether circC6orf132 was related to glycolysis *in vivo*, we detected the production of lactate and the uptake of glucose in the serum of mice. As exhibited in Figures 8F,G, lactate production and glucose uptake were significantly reduced in sh-circC6orf132 group compared with sh-NC group. Additionally, IHC analysis demonstrated that GLUT1 and HK2 protein levels were downregulated in tumor tissues of sh-circC6orf132 group relative to sh-NC group (Figure 8H). IHC assay also demonstrated that the protein levels of proliferation-associated Ki-67 and PCNA were reduced by silence of circC6orf132 (Figure 8I). Tumor growth and glycolysis were also retarded by knockdown of circC6orf132 *in vivo* through the mediation of miR-873-5p and PRKAA1. Overall, circC6orf132 downregulation inhibited the GC progression *in vitro* and *in vivo* through regulating the miR-873-5p/PRKAA1 axis.

## Discussion

CircRNAs are one of the most common RNA regulatory molecules in cancer research, including GC (Zhang and Du, 2016). Nevertheless, circRNA reports in GC under hypoxia are few. Herein, we found that circC6orf132 affected the glycolytic metabolism and malignant progression of GC cells under hypoxia. Meanwhile, its mechanism of action was also been clarified.

Hypoxia is one of the pivotal features of solid tumors. Hypoxic microenvironment can promote tumor development and confer chemo/radioresistance on tumor cells through affecting cellular metabolism and exerting molecular responses (Joseph et al., 2018). Through detecting the expression of HIF1α, we have identified the hypoxia environment in GC cells. Then, GC cell proliferation, migration, and invasion were all promoted under hypoxia condition. Glycolytic metabolism is accompanied by the uptake of large glucose, the production of lactate, and ATP under hypoxia (de Bari and Atlante, 2018). Our results indicated that lactate accumulation, glucose uptake, ATP production, and ECAR were all increased in hypoxic GC cells compared with normoxic GC cells. Glycolytic metabolism depends on the involvement of GLUT1 for glucose transport and HK2 for glucose phosphorylation (Massari et al., 2016). Western blotting also revealed that GLUT1 and HK2 protein levels were significantly upregulated by hypoxia, suggesting that hypoxia indeed facilitated the process of glycolysis in GC cells.

CircRNAs have been demonstrated to be correlated to the biological effect of hypoxia on GC cells. For example, the hypoxia-induced migration and invasion of GC cells were weakened after downregulating circ_0083143 (Tang et al., 2020). CircNRIP1 was found to enhance the hypoxia-induced chemoresistance in GC by affecting glycolysis (Xu et al., 2020). In this chapter, circC6orf132 was upregulated in GC cells under hypoxia. Functionally, knockdown of circC6orf132 lightened the promoting effects on GC cell proliferation, migration, invasion, and glycolysis caused by hypoxia. Meanwhile, upregulation of circC6orf132 enhanced the malignant behaviors in hypoxic GC cells. Thus, the role of hypoxia in GC cell progression and glycolysis relied on the upregulation of circC6orf132 partly.

CircRNAs are known as the molecular sponges for varied miRNAs to affect their function in cancers (Panda, 2018). In GC research, circRNA0047905 acted as a tumor promoter by the sponge effects on miR-4516 and miR-1227-5p (Lai et al., 2019), and circNF1 motivated GC cell proliferation by absorbing miR-16 (Wang et al., 2019). In addition, circRHOBTB3 and cirMC3 generated the anti-tumor responses on GC *via*, respectively, sponging miR-654-3p and miR-296-5p (Rong et al., 2019; Deng et al., 2020). As for the miRNA sponge function of circC6orf132, we have validated the binding between circC6orf132 and miR-873-5p in GC cells and found that miR-873-5p inhibitor rescued the si-circC6orf132-aroused regulation in GC cells under hypoxia. Evidently, the function of circC6orf132 was associated with its sponge mechanism of miR-873-5p.

Subsequently, miR-873-5p was exhibited to target the 3′UTR to downregulate the expression of PRKAA1 in GC cells. PRKAA1 is a submember of AMPK family, which has vital effects on hypoxia-induced diseases (Huang et al., 2014; Gui et al., 2017; Owada et al., 2018). Our data showed that PRKAA1 was overexpressed in hypoxic GC cells, and its overexpression abated the inhibition of miR-873-5p for hypoxia-induced GC progression and glycolysis promotion. These results identified that PRKAA1 was as an oncogene in GC, and miR-873-5p played an inhibitory role in GC by reducing PRKAA1 expression. What is more, circC6orf132 affected PRKAA1 level *via* the interaction with miR-873-5p. *In vivo* assays further manifested the presence of circC6orf132/miR-873-5p/PRKAA1 axis in regulating tumorigenesis and glycolysis of GC. Meanwhile, this regulatory axis was validated in the regulation of malignant progression of GC cells under normoxia.

## Conclusion

**FIGURE 9:**
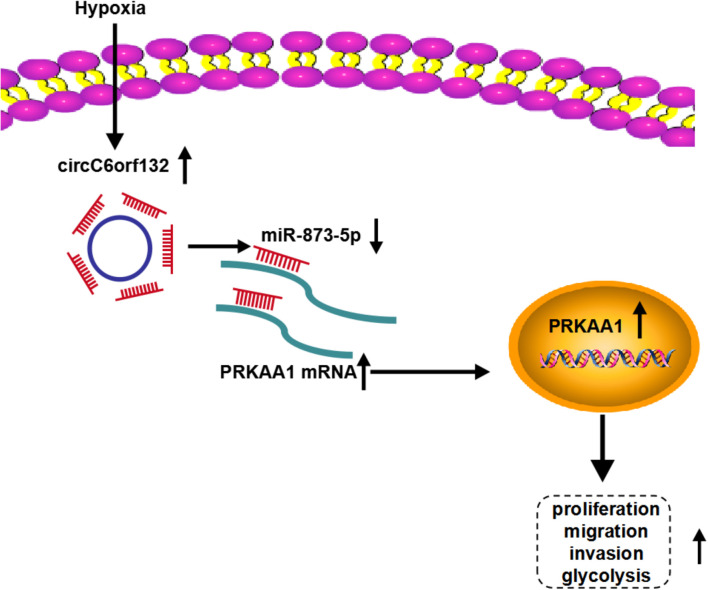
Schematic diagram of circC6orf132/miR-873-5p/PRKAA1 in hypoxia-induced gastric cancer (GC) progression.

This research elucidated that circC6orf132 acted as a miR-873-5p sponge to upregulate the mRNA and protein levels of PRKAA1, thus promoting the malignant behaviors of GC cells under hypoxia environment (Figure 9). CircC6orf132 was implicated in the hypoxia-induced GC progression and glycolytic metabolism, and its pathological mechanism was found firstly in our study. All these findings afforded a specific circC6orf132/miR-873-5p/PRKAA1 regulatory network for GC development under hypoxia or normoxia.

## Data Availability Statement

The raw data supporting the conclusions of this article will be made available by the authors, without undue reservation.

## Ethics Statement

The studies involving human participants were reviewed and approved by The First Affiliated Hospital of Jinzhou Medical University. The patients/participants provided their written informed consent to participate in this study.

## Author Contributions

WC: conception and design of study, perform the experiments, manuscript writing, and project supervision. YJ: data analysis and interpretation, conception and design of study, manuscript writing, and project supervision.

## Conflict of Interest

The authors declare that the research was conducted in the absence of any commercial or financial relationships that could be construed as a potential conflict of interest.

## Publisher’s Note

All claims expressed in this article are solely those of the authors and do not necessarily represent those of their affiliated organizations, or those of the publisher, the editors and the reviewers. Any product that may be evaluated in this article, or claim that may be made by its manufacturer, is not guaranteed or endorsed by the publisher.

## Supplementary Material

Supplementary Figure 1 Hypoxia induced the upregulation of HIF1α in GC cells. The protein level of HIF1α was detected by western blot after AGS and HGC-27 cells were treated with hypoxia. ^∗∗^*P* < 0.01, ^∗∗∗^*P* < 0.001.

Supplementary Figure 2 Overexpression of circC6orf132 enhanced the hypoxia-induced tumor progression in GC cells. AGS and HGC-27 cells were transfected with vector or circC6orf132 under hypoxia for 48 h, and untransfected cells were cultured under normoxia or hypoxia for 48 h. **(A)** CircC6orf132 level was assayed through qRT-PCR. **(B–D)** The proliferation **(B)** and migration or invasion **(C,D)** were assessed *via* colony formation assay and transwell assay. **(E–G)** Glycolysis was analyzed through lactate production **(E)**, glucose uptake **(F)** and ATP level **(G)**. ^∗∗^*P* < 0.01, ^∗∗∗^*P* < 0.001, and ^****^*P* < 0.0001.

Supplementary Figure 3 The absolute quantification of circC6orf132 and miR-873-5p in normal and GC tissues. **(A,B)** Standard curves of circC6orf132 and miR-873-5p. **(C)** Copy number of circC6orf132 and miR-873-5p in GC tissues. ^****^*P* < 0.0001.

Supplementary File 1 The original pictures of Figures 2, 4, 6, 8, and Supplementary Figure 2.
