# Supplementary material for: CircC6orf132 Facilitates Proliferation, Migration, Invasion, and Glycolysis of Gastric Cancer Cells Under Hypoxia by Acting on the miR-873-5p/PRKAA1 Axis
Source: Front Genet. 2021 Sep 30;12:636392. doi: 10.3389/fgene.2021.636392 (PMC8514671; doi:10.3389/fgene.2021.636392)

Supplementary Fig.1

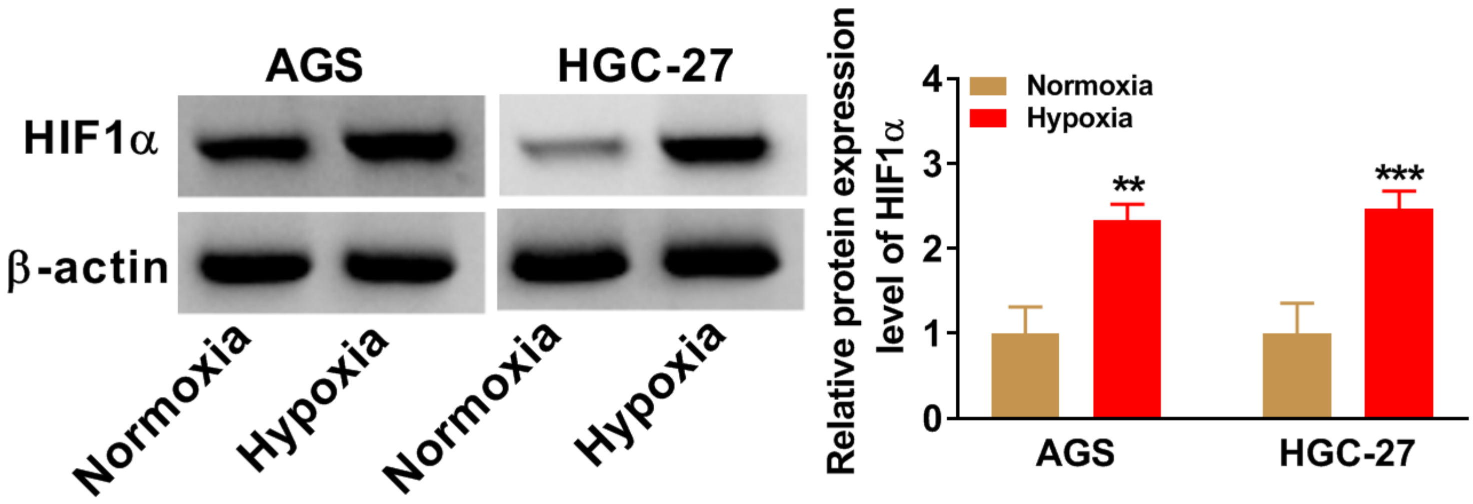

Supplementary Fig.2

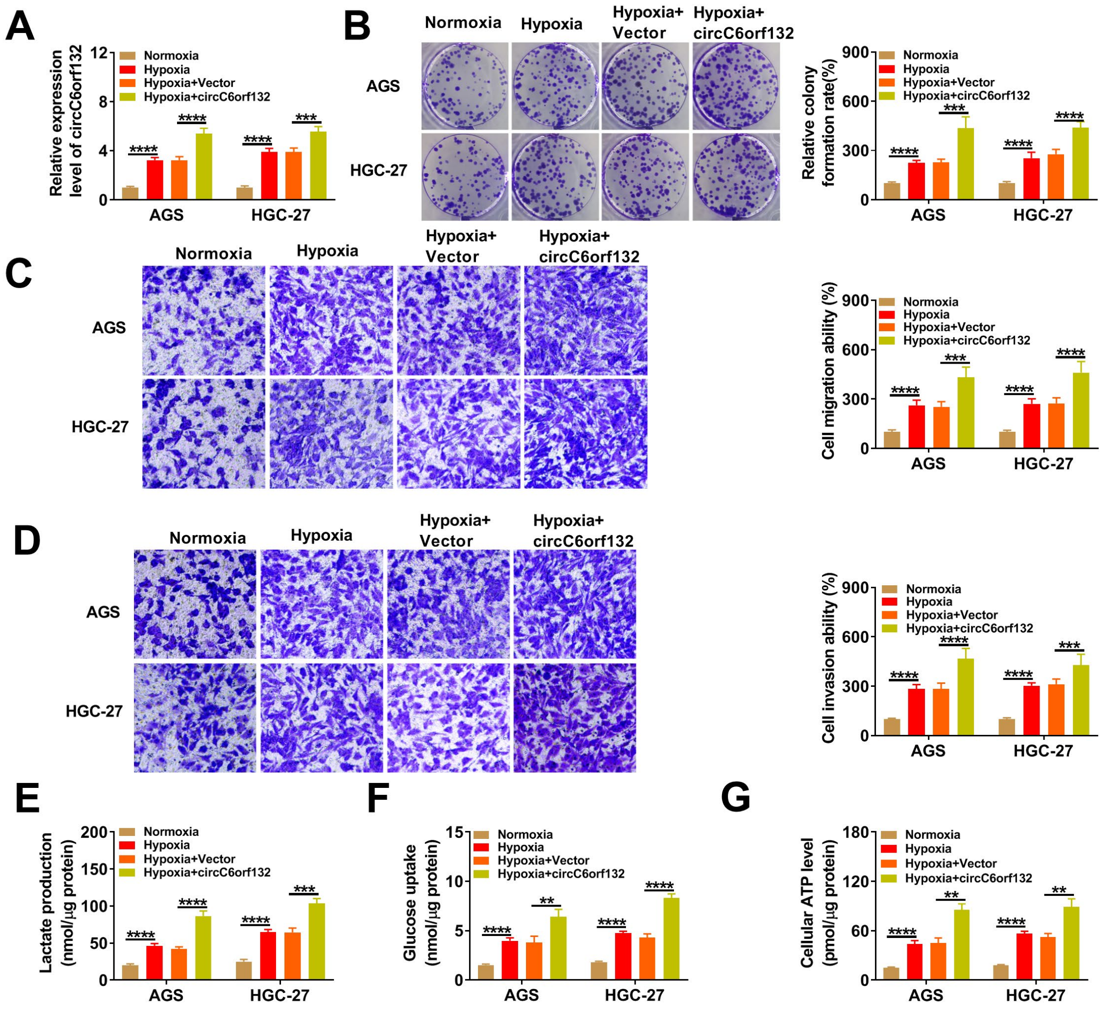

Supplementary Fig.3

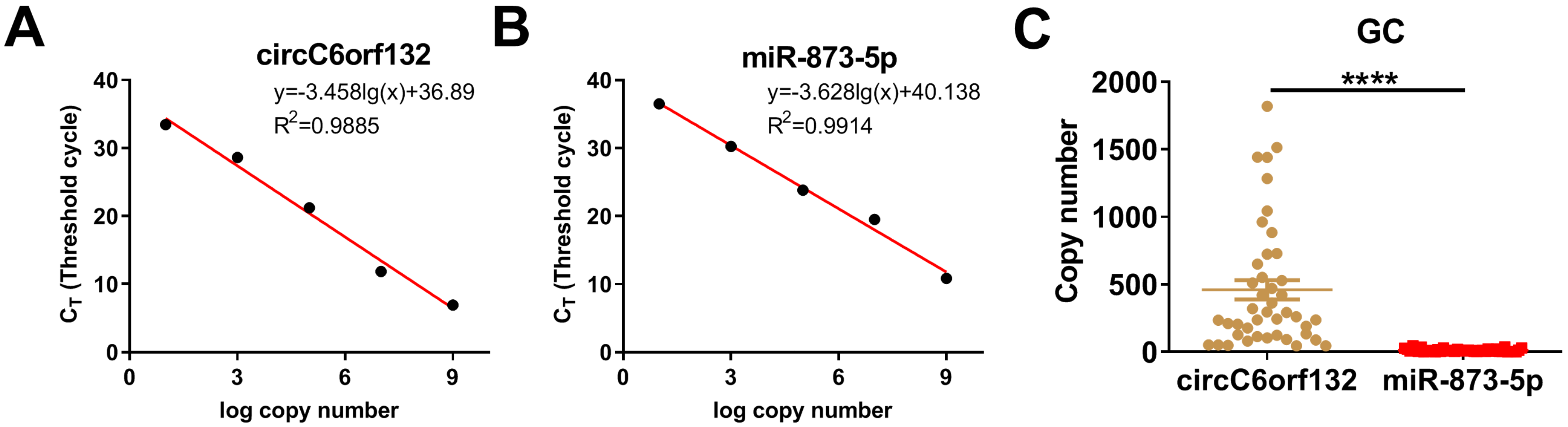

Supplementary file 1
(The original pictures of Fig. 2, Fig. 4, Fig. 6, Fig. 8 and Supplementary Fig. 2.)

Fig.2B

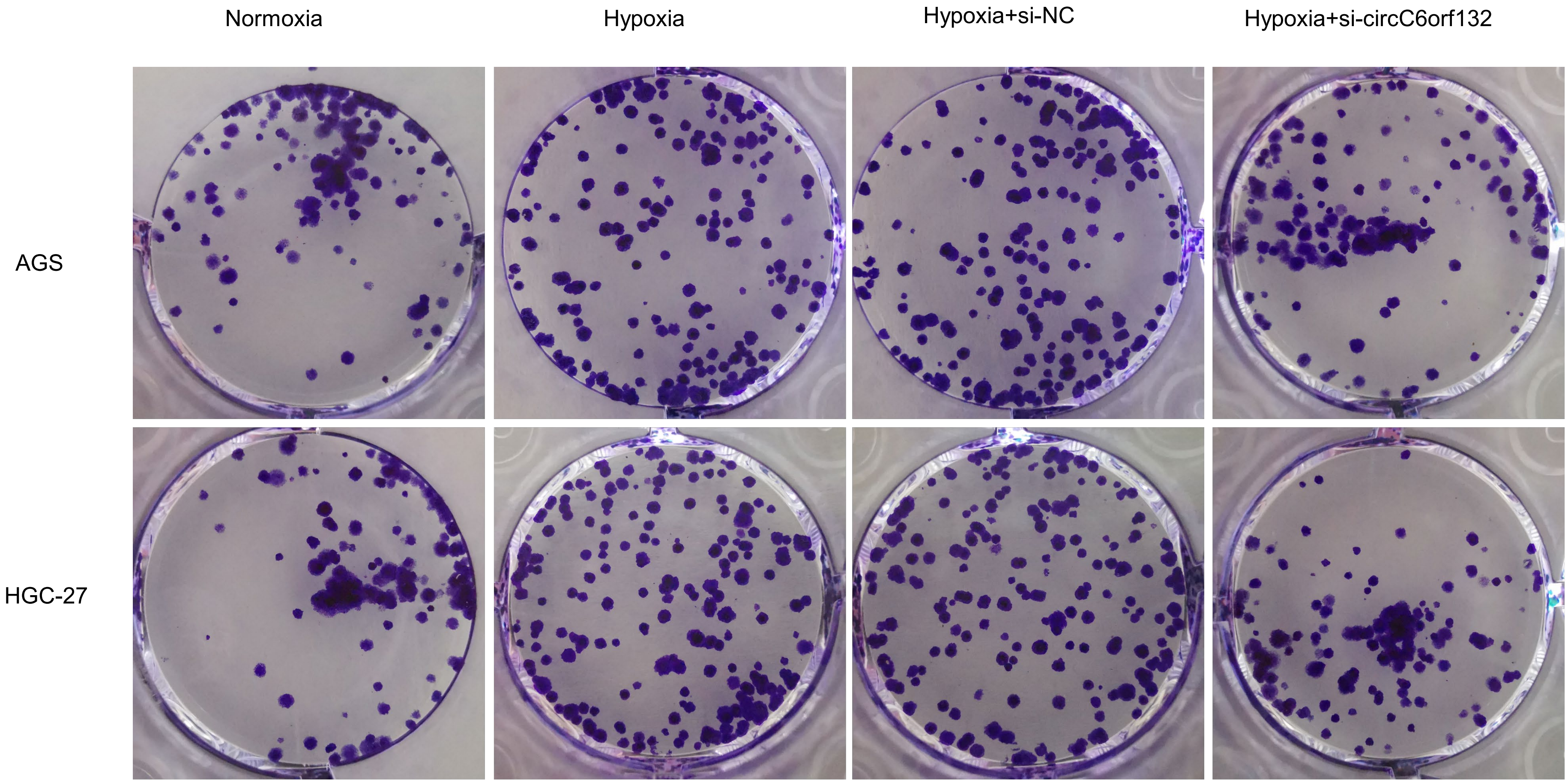

Fig.2C

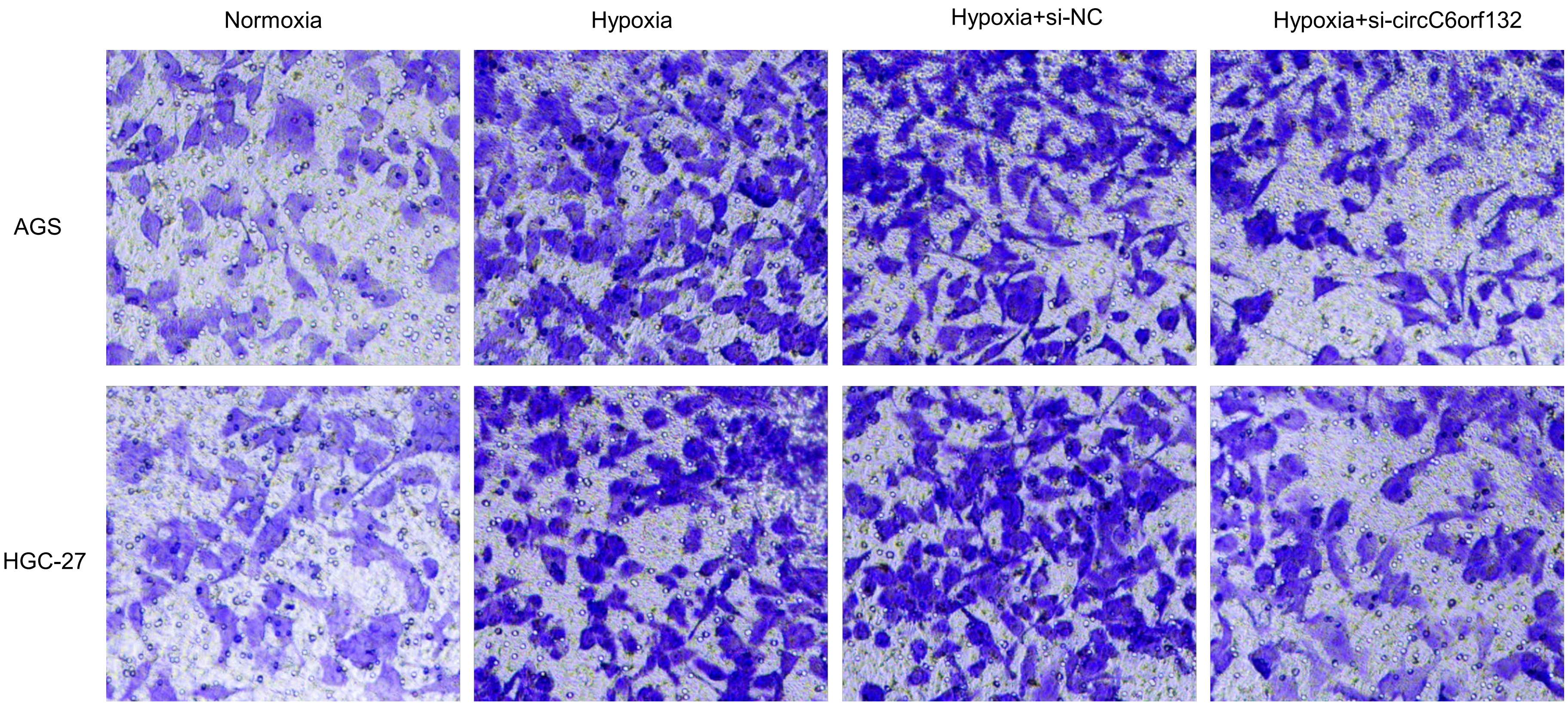
Normoxia
Hypoxia
Hypoxia+si-NC
Hypoxia+si-circC6orf132
AGS
HGC-27

Fig.2D

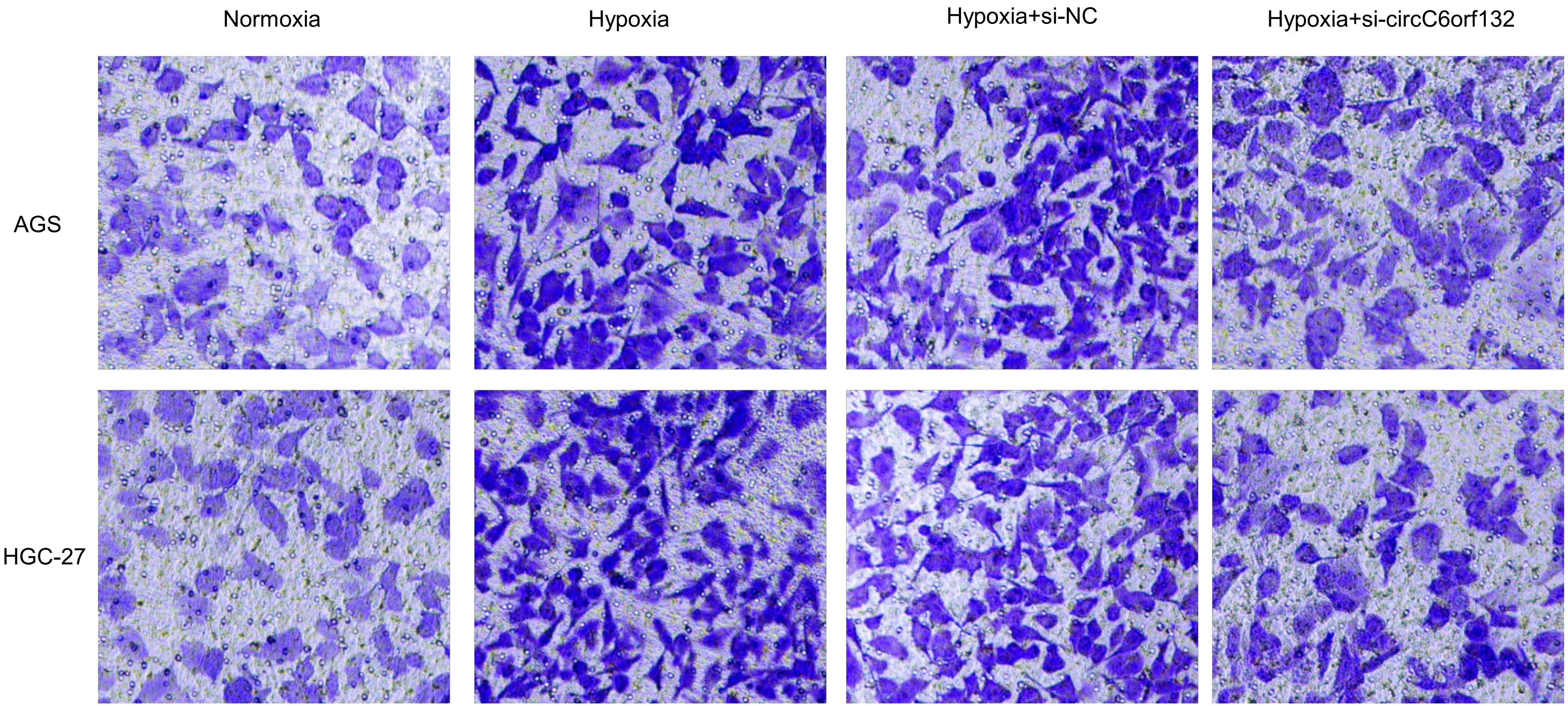
Normoxia
Hypoxia
Hypoxia+si-NC
Hypoxia+si-circC6orf132
AGS
HGC-27

Fig.4B

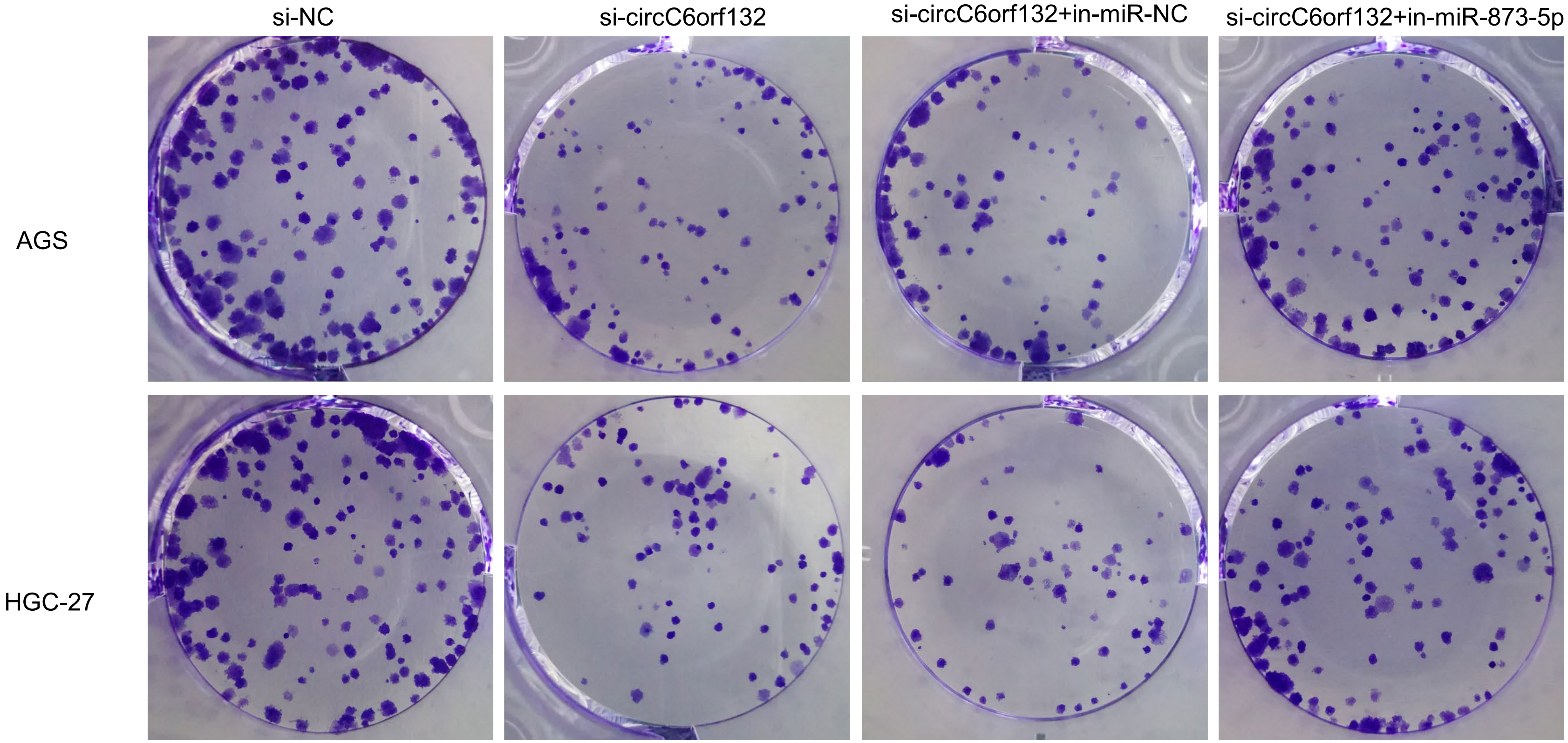
si-NC
si-circC6orf132
si-circC6orf132+in-miR-NC
si-circC6orf132+in-miR-873-5p
AGS
HGC-27

Fig.4C

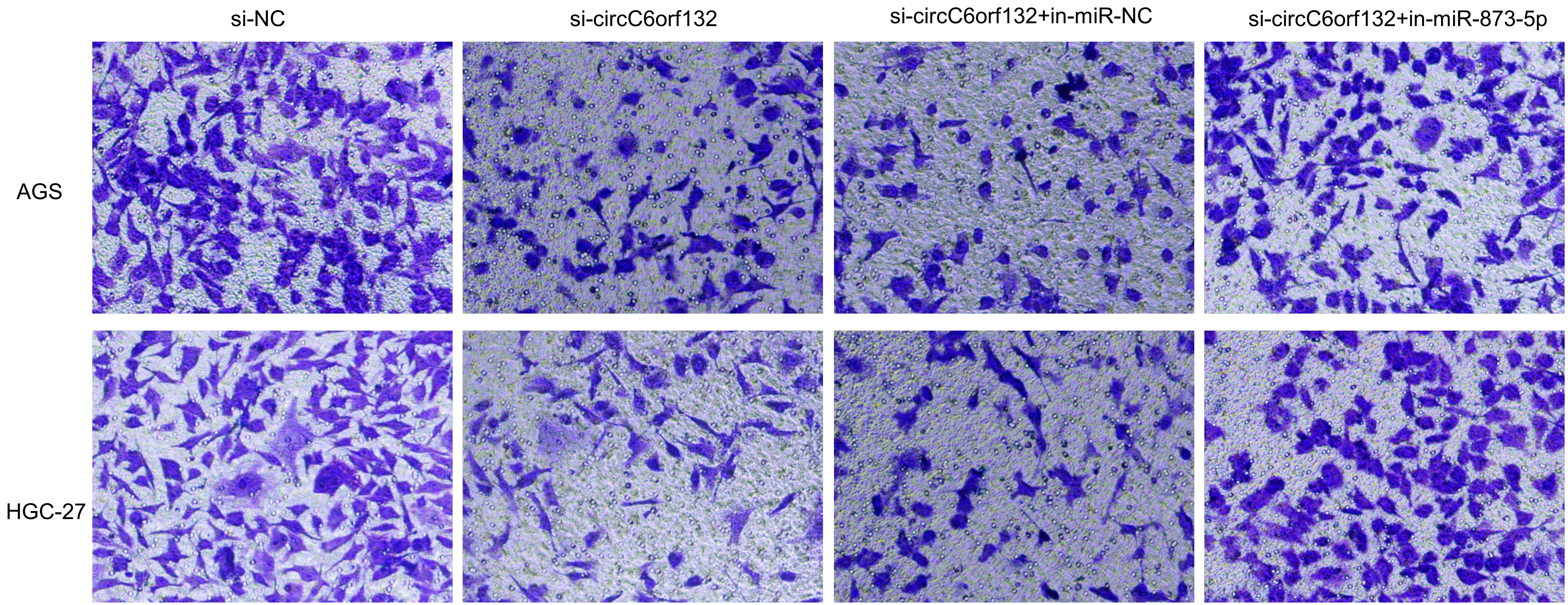

si-NC
si-circC6orf132
si-circC6orf132+in-miR-NC
si-circC6orf132+in-miR-873-5p
AGS
HGC-27

Fig.4D

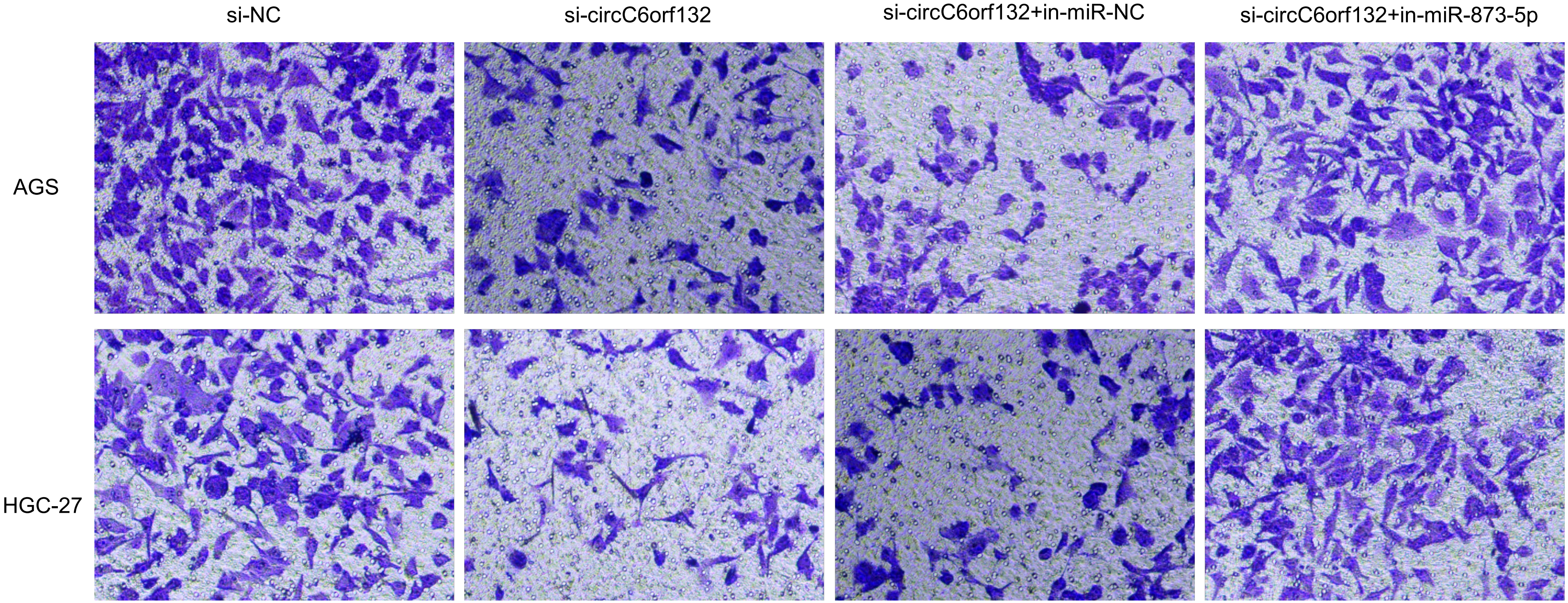
si-NC
si-circC6orf132
si-circC6orf132+in-miR-NC
si-circC6orf132+in-miR-873-5p
AGS
HGC-27

Fig.6B

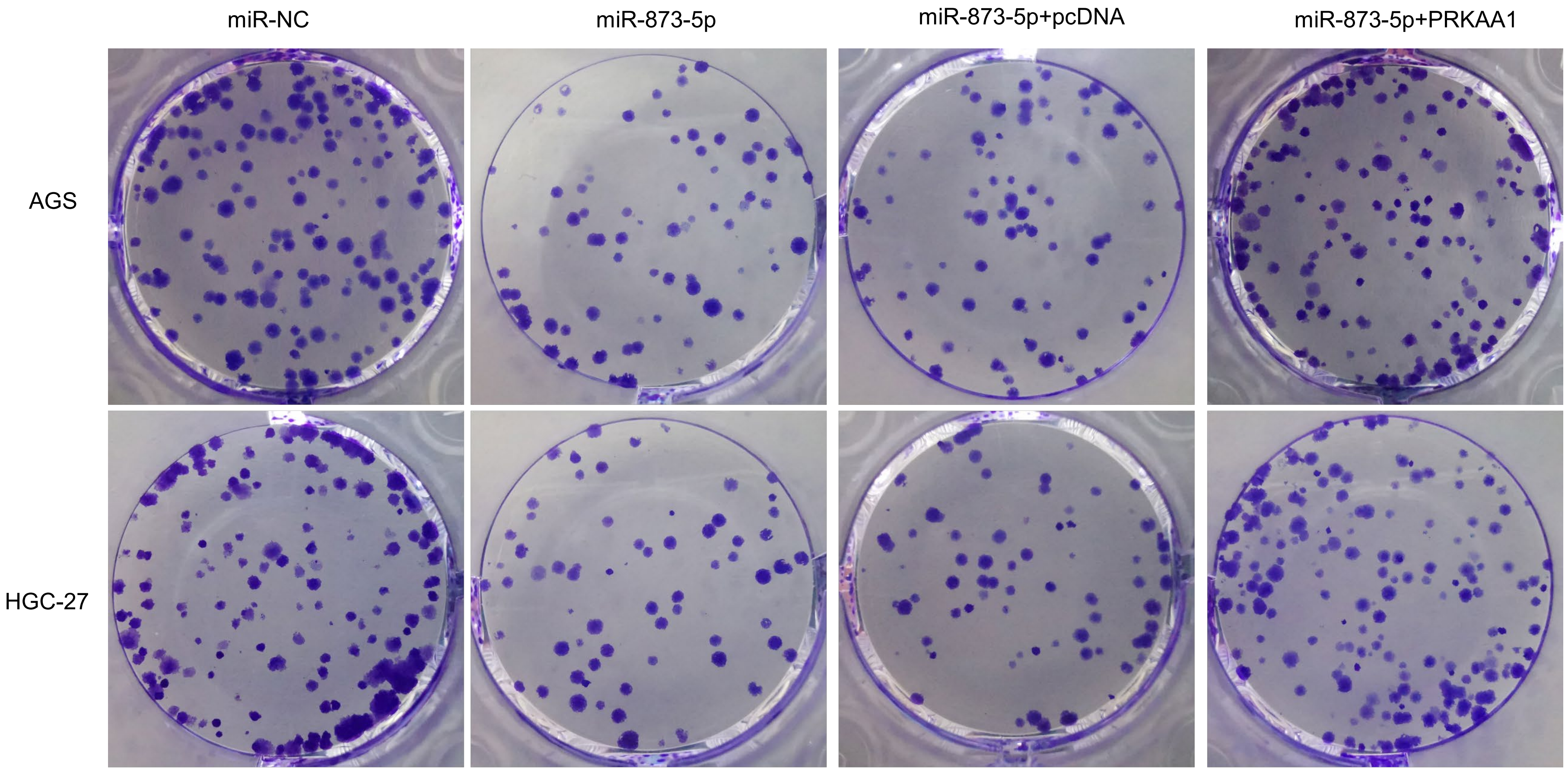

Fig.6C

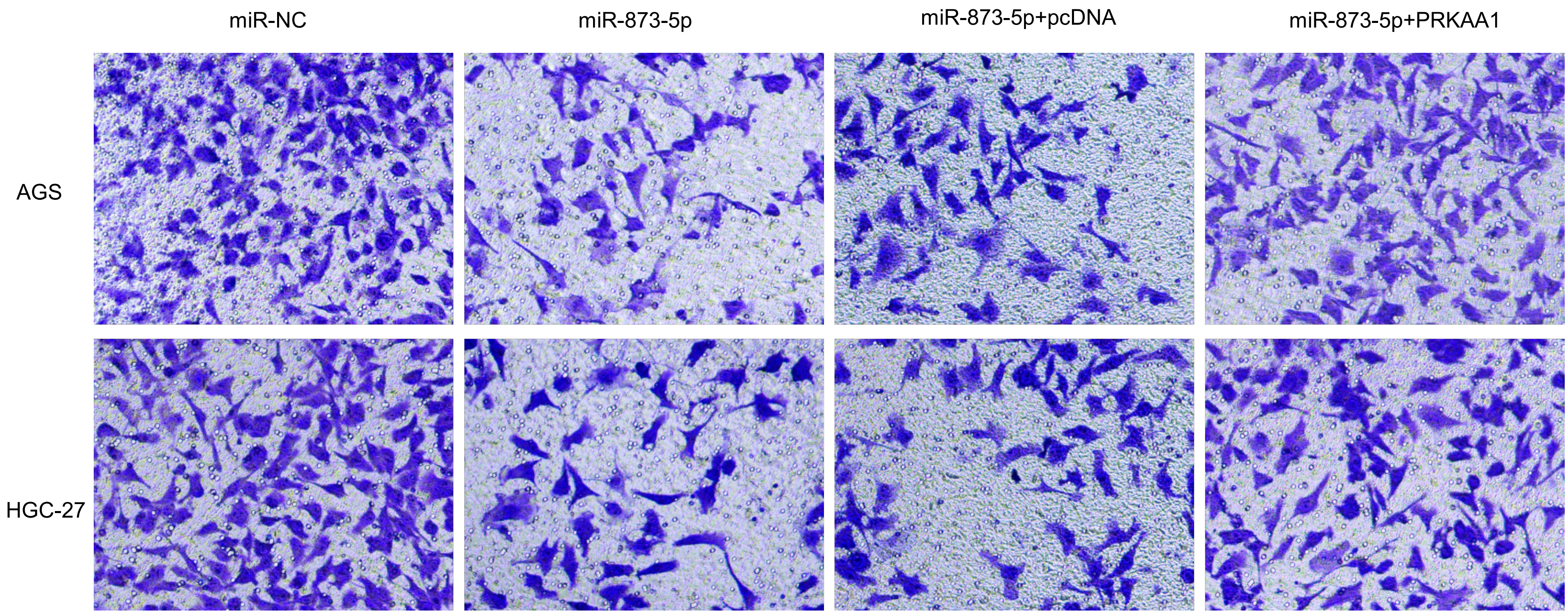

Fig.6D

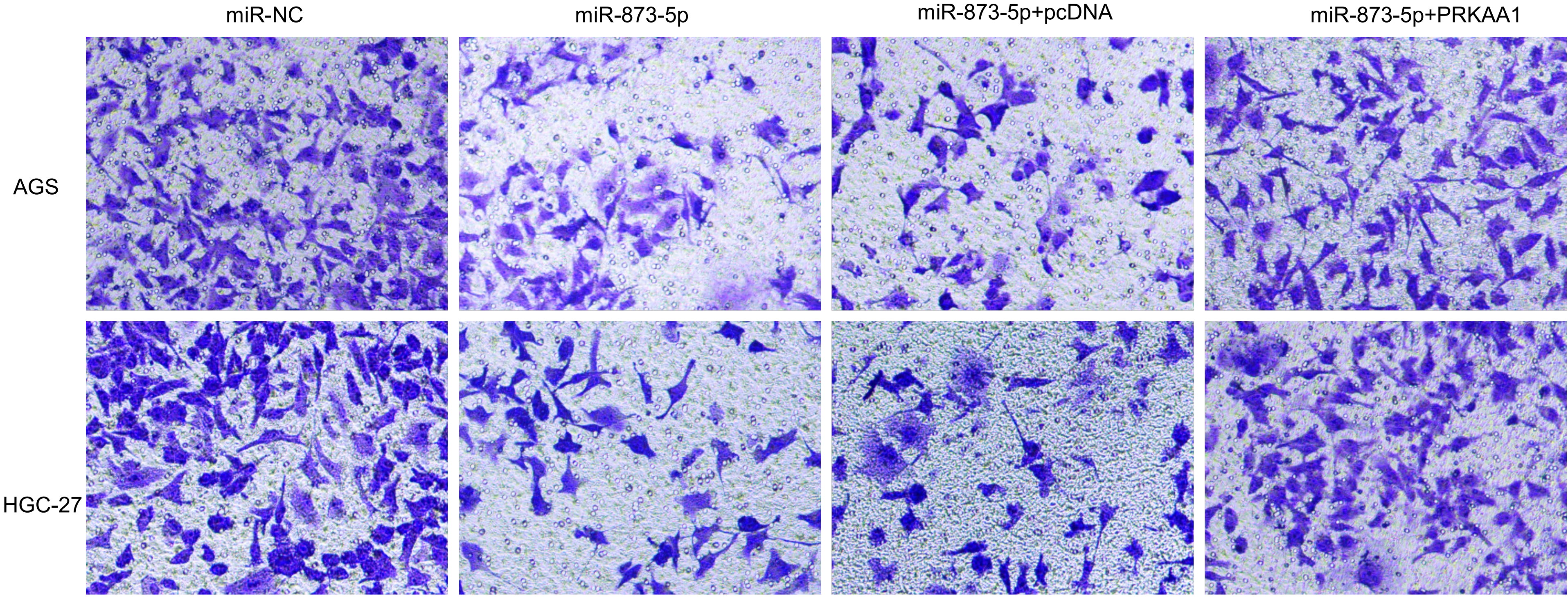

Fig.7A

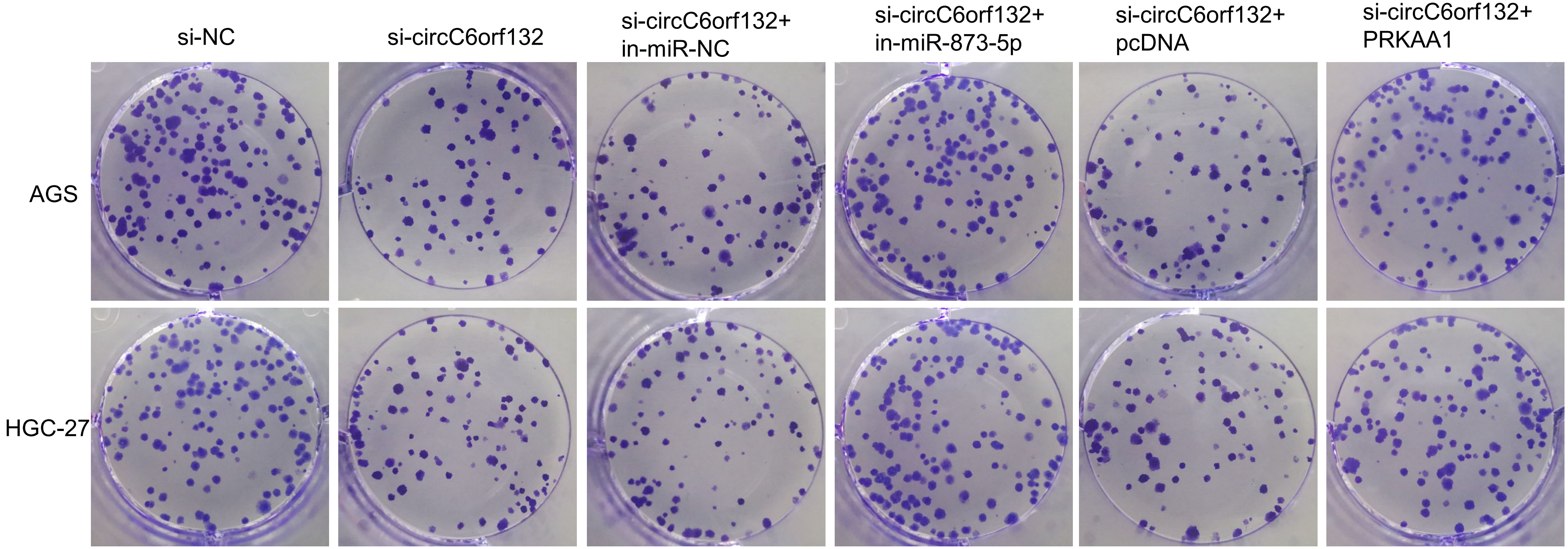
si-NC
si-circC6orf132
si-circC6orf132+
in-miR-NC
si-circC6orf132+
in-miR-873-5p
si-circC6orf132+
pcDNA
si-circC6orf132+
PRKAA1
AGS
HGC-27

Fig.7B

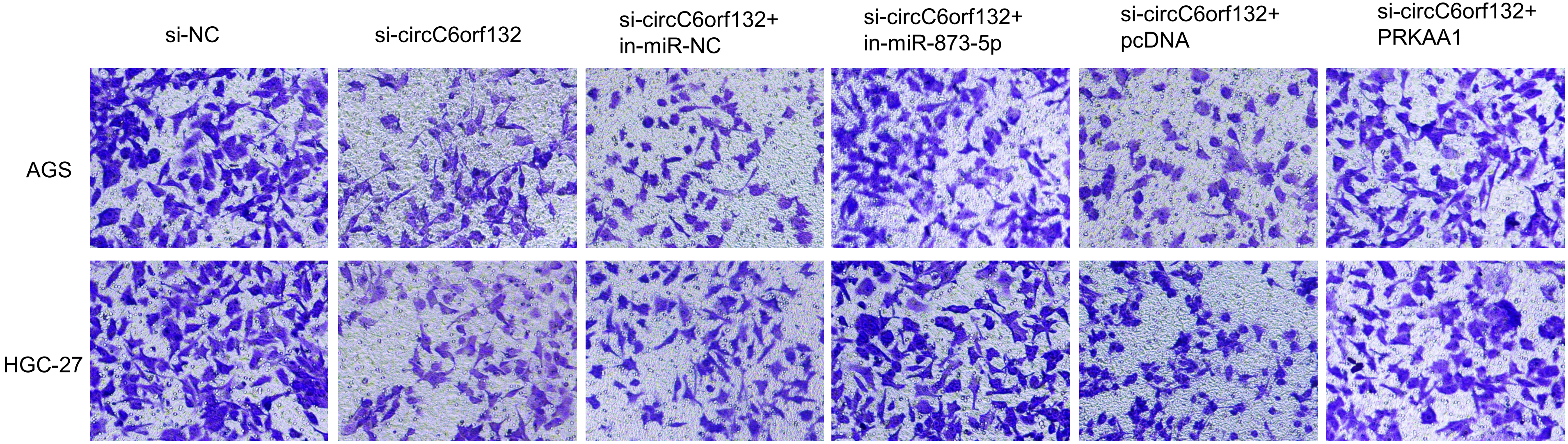
si-NC
si-circC6orf132
si-circC6orf132+ in-miR-NC
si-circC6orf132+ in-miR-873-5p
si-circC6orf132+ pcDNA
si-circC6orf132+ PRKAA1
AGS
HGC-27

Fig.7C

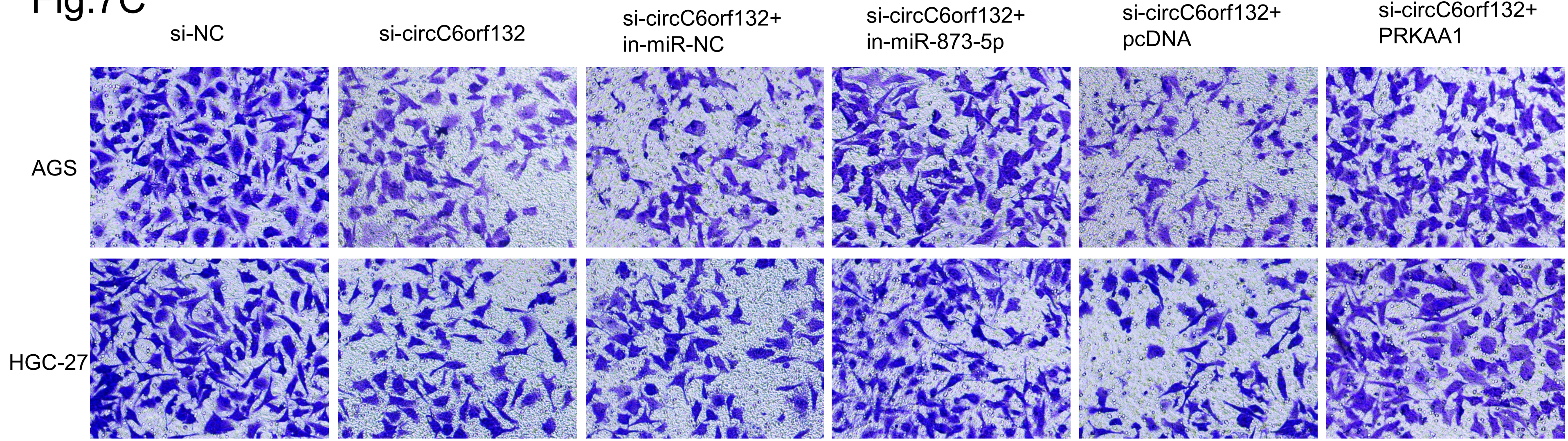
si-NC
si-circC6orf132
si-circC6orf132+ in-miR-NC
si-circC6orf132+ in-miR-873-5p
si-circC6orf132+ pcDNA
si-circC6orf132+ PRKAA1
AGS
HGC-27

Supplementary Fig.2B

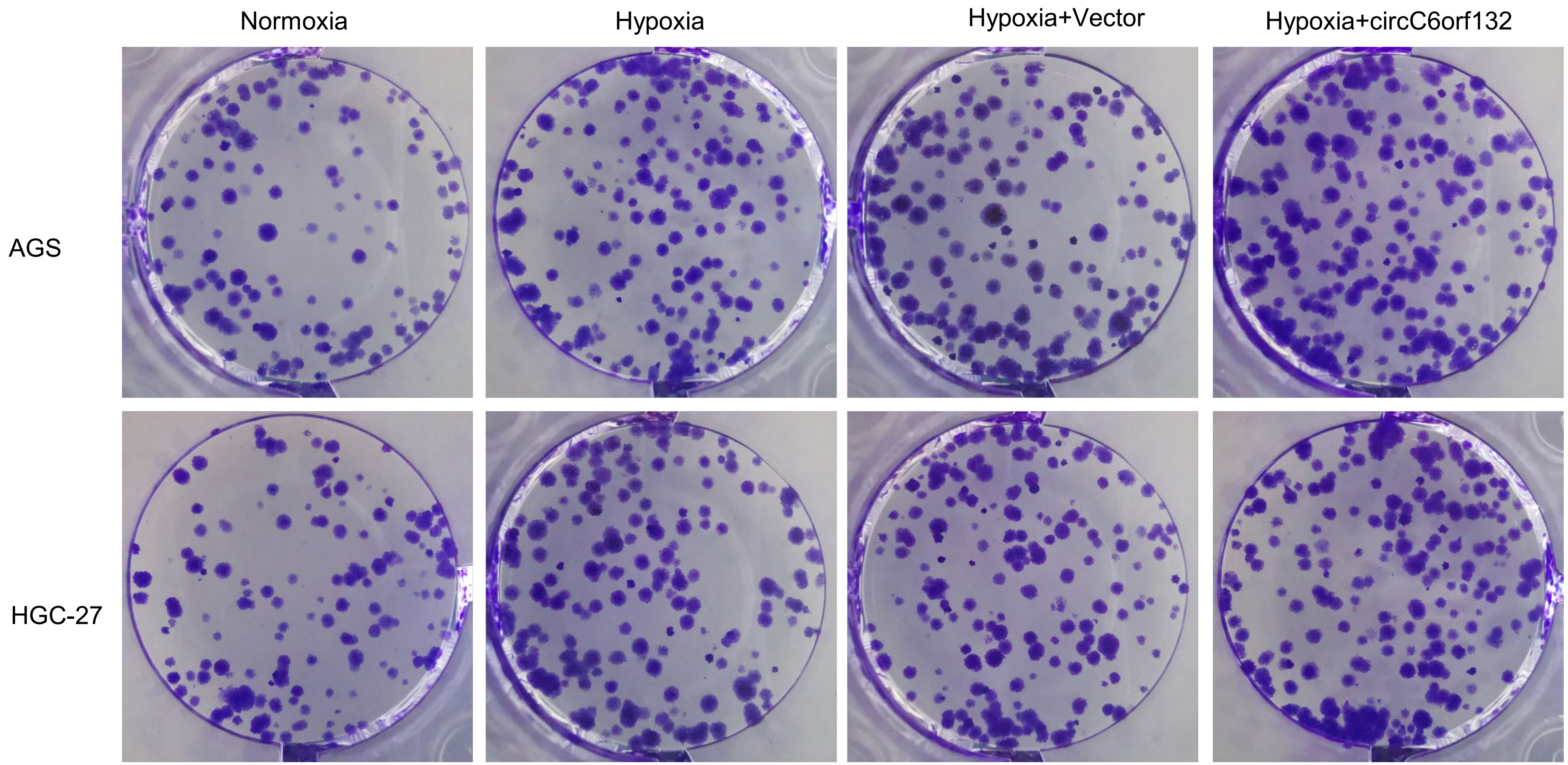

Supplementary Fig.2C

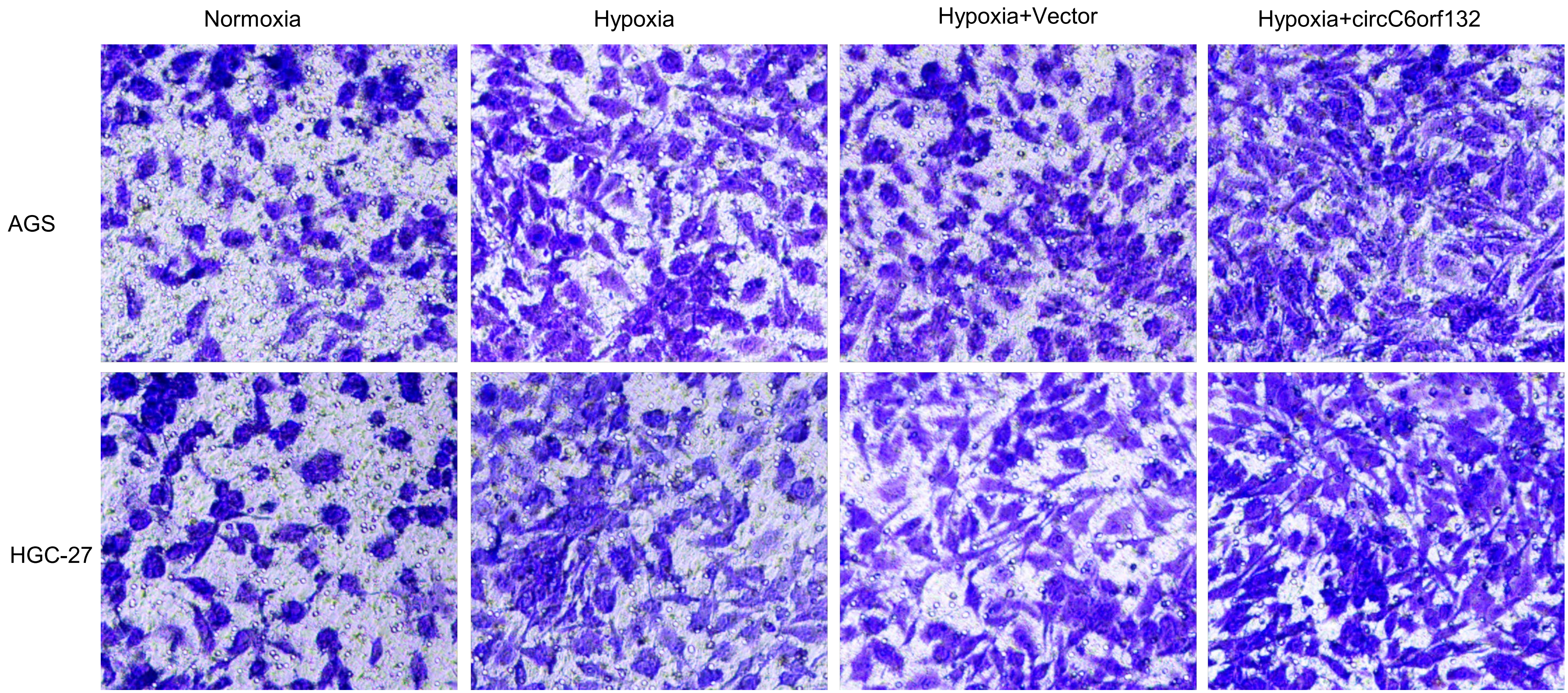

Supplementary Fig.2D

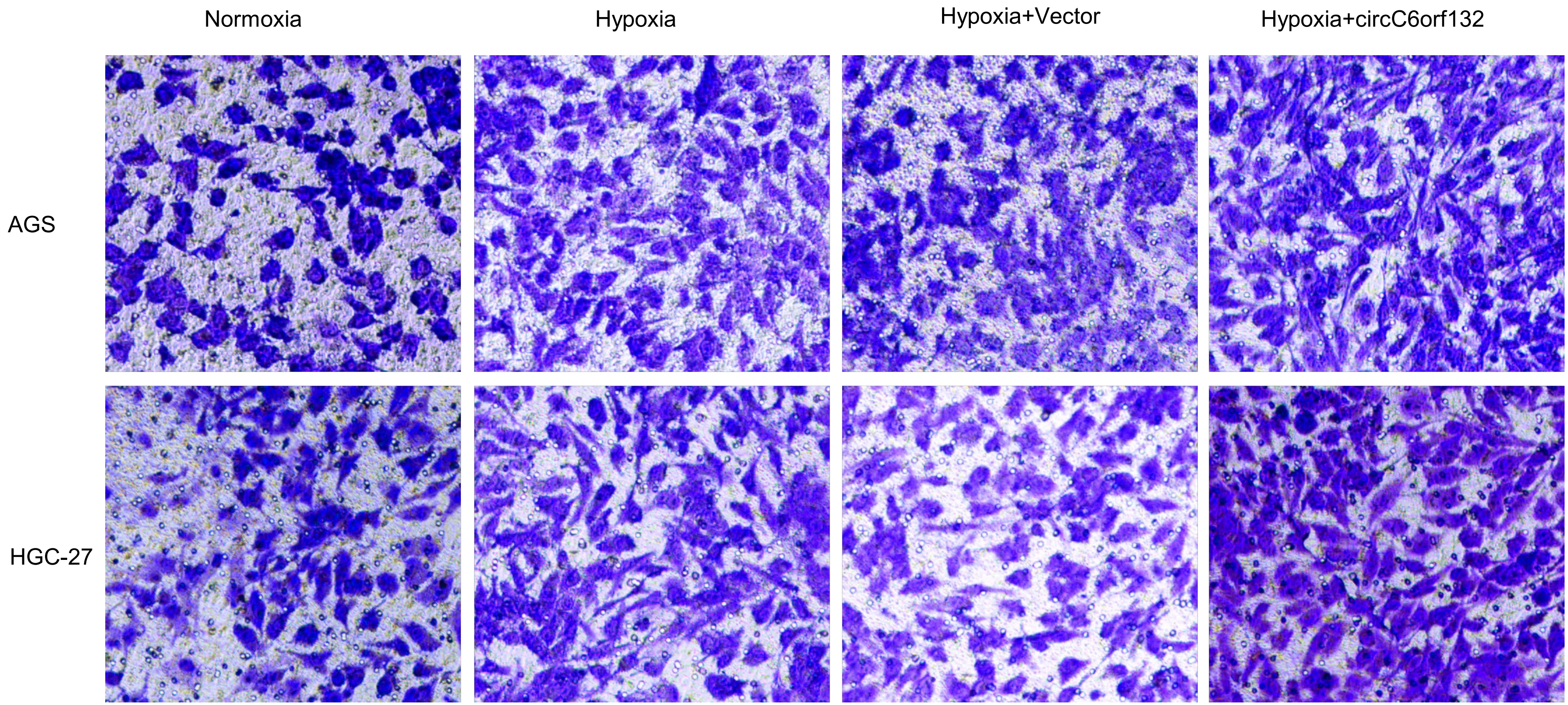

Fig.8B

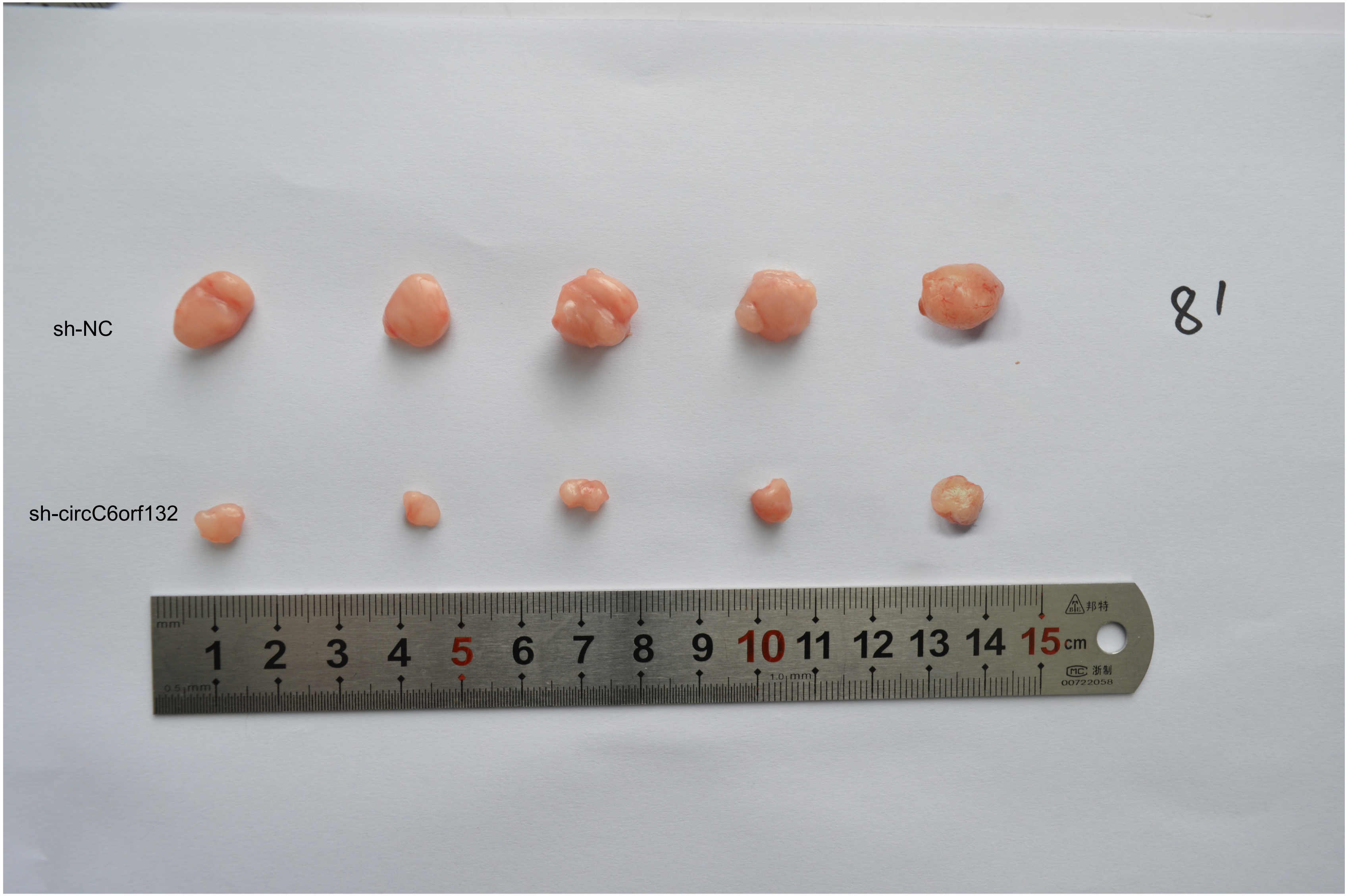

Supplement: Supplementary Figure 1 — Hypoxia induced the upregulation of HIF1α in GC cells. The protein level of HIF1α was detected by western blot after AGS and HGC-27 cells were treated with hypoxia. ∗∗P < 0.01, ∗∗∗P < 0.001. [file Data_Sheet_1.pdf]
